# Optical Genome Mapping Reveals the Complex Genetic Landscape of Myeloma

**DOI:** 10.3390/cancers15194687

**Published:** 2023-09-22

**Authors:** Amélie Giguère, Isabelle Raymond-Bouchard, Vanessa Collin, Jean-Sébastien Claveau, Josée Hébert, Richard LeBlanc

**Affiliations:** 1 Cytogenetics Laboratory, Maisonneuve-Rosemont Hospital, Montreal, QC H1T 2M4, Canada; isabelle.raymond-bouchard.cemtl@ssss.gouv.qc.ca (I.R.-B.); vanessa.collin.cemtl@ssss.gouv.qc.ca (V.C.); josee.hebert@umontreal.ca (J.H.); 2 Division of Hematology, Oncology and Transplantation, Department of Medicine, Maisonneuve-Rosemont Hospital, Université de Montréal, Montreal, QC H1T 2M4, Canada; jean-sebastien.claveau.med@ssss.gouv.qc.ca (J.-S.C.); richard.leblanc.med2@ssss.gouv.qc.ca (R.L.)

**Keywords:** myeloma, optical genome mapping, cytogenetics, *IGH* translocations, hyperdiploidy, *PVT1* rearrangement, 1p deletion, 1q gain/amplification

## Abstract

**Simple Summary:**

Myeloma is a hematological cancer characterized by numerous genetic abnormalities currently identified using fluorescent in situ hybridization (FISH). However, FISH is a targeted method offering a limited view of the genome mainly restricted to chromosomal translocations involving the *IGH* gene and aberrations of chromosomes 1 and 17. A novel technology called optical genome mapping (OGM) has the potential to overcome these limitations. The goal of our study was to evaluate if OGM could replace FISH as a standard method for detection of these abnormalities. We performed OGM analysis on 20 myeloma patients and found that OGM could detect all the classic abnormalities in myeloma. OGM identified additional abnormalities across the entire genome and provided information on their larger genomic context. To our knowledge, this is the first study to show the potential of OGM to significantly change how we detect and study abnormalities in myeloma in a clinical context.

**Abstract:**

Fluorescence in situ hybridization (FISH) on enriched CD138 plasma cells is the standard method for identification of clinically relevant genetic abnormalities in multiple myeloma. However, FISH is a targeted analysis that can be challenging due to the genetic complexity of myeloma. The aim of this study was to evaluate the potential of optical genome mapping (OGM) to detect clinically significant cytogenetic abnormalities in myeloma and to provide larger pangenomic information. OGM and FISH analyses were performed on CD138-purified cells of 20 myeloma patients. OGM successfully detected structural variants (SVs) (*IGH* and *MYC* rearrangements), copy number variants (CNVs) (17p/*TP53* deletion, 1p deletion and 1q gain/amplification) and aneuploidy (gains of odd-numbered chromosomes, monosomy 13) classically expected with myeloma and led to a 30% increase in prognosis yield at our institution when compared to FISH. Despite challenges in the interpretation of OGM calls for CNV and aneuploidy losses in non-diploid genomes, OGM has the potential to replace FISH as the standard of care analysis in clinical settings and to efficiently change how we identify prognostic and predictive markers for therapies in the future. To our knowledge, this is the first study highlighting the feasibility and clinical utility of OGM in myeloma.

## 1. Introduction

Multiple myeloma (MM) is a plasma cell neoplasm associated with a high burden of morbidity and remains incurable in the vast majority of cases [1]. MM progresses from pre-malignant states (monoclonal gammopathy of undetermined significance and smoldering myeloma) to a more aggressive disease over time, with the acquisition of numerous genetic alterations. These include structural variations (e.g., balanced and unbalanced translocations, insertions or inversions), copy number variations (e.g., deletions/losses and duplications/gains), aneuploidies (e.g., trisomies, monosomies) and mutations [2].

Structural variations (SVs) have been known to play an important role in the genesis and development of MM for more than 20 years and cytogenetics has been and remains the gold standard in MM testing for SV identification. Early studies focusing on karyotyping of MM provided insights into the complexity of MM genomes revealing two major categories of genetic abnormalities detected at disease initiation. These arehyperdiploid myeloma (HDM) with gains of odd-numbered chromosomes [3] and non-hyperdiploid genomes with translocations of the *IGH* gene leading to overexpression of oncogenes through superenhancer hijacking—*NSD2,* previously known as *MMSET* (t(4;14)(p16;q32)), *MAF* (t(14;16)(q32;q23)), *MAFB* (t(14;20)(q32;q12)), *CCND1* (t(11;14)(q13;q32)) and *CCND3* (t(6;14)(p21;q32)) [4]. Copy number variations (CNVs) leading to 1p32 or 17p13 deletion and 1q21 gain/amplification as well as 8q24/*MYC* abnormalities are considered secondary abnormalities involved in disease progression [5,6].

In recent years, a number of studies using high-throughput next-generation sequencing (NGS) technologies have revealed numerous gene variants (e.g., *KRAS*, *NRAS*, *FAM46C*, *DIS3* and *TP53)* [7] and have provided a better understanding of the identity and composition of SVs that make up the genomes of newly diagnosed and relapsed MM [8,9]. These studies have also shed light on the mechanisms that likely contribute to the emergence of these SVs, confirming that MM genomes are composed of both multiple simple aberrations as well as highly complex SVs that are the result of potentially more complex oncogenic mechanisms [10]. However, except for *TP53* biallelic inactivation, which was recently introduced into clinical guidelines, these more recently discovered SVs have yet to be included in large-scale prognostic studies, in part due to the lack of efficient methods for detection of these abnormalities in clinical settings. This limitation hinders the ability to fully evaluate the genomic complexity of MM and to identify the potential prognostic and predictive impact of these abnormalities, in addition to preventing the development of novel treatment options. Novel methods are needed in clinical laboratories to allow for faster and more conclusive identification of SVs in MM, which, in turn, would allow these SVs to be more readily included in larger-scale prognostic studies.

In clinical practice, prognostication of MM using the revised International Staging System (R-ISS) [11] or the second revision of the International Staging System (R2-ISS) [12] is based solely on three high-risk cytogenetic abnormalities: t(4;14), t(14;16) and 17p/*TP53* deletion for R-ISS and t(4;14), 17p/*TP53* deletion and 1q21 gain/amplification for R2-ISS, in addition to biochemical markers including β2-microglobulin, serum lactate dehydrogenase and serum albumin. Fluorescence in situ hybridization (FISH) on CD138-purified plasma cells, minimally targeting 17p/*TP53* deletions, t(4;14), t(11;14) and t(14;16) translocations as well as chromosome 1 abnormalities, represents the most widely available and accepted method in clinical practice for SV identification in MM [12,13]. However, other clinically relevant abnormalities which have been shown to be high-risk independent markers should also be investigated, such as t(14;20) and 8q24.21/*MYC* rearrangements [12,14,15]. Moreover, MM patients with t(11;14) have distinct features, including cyclin D1 expression, high levels of BCL2 expression and CD20 B-cell lineage protein expression. The prognostic impact of t(11;14) is currently being discussed in the era of novel therapies, and this cytogenetic abnormality is considered as a novel predictive marker in MM as patients with t(11;14) are sensitive to venetoclax, a highly selective inhibitor of BCL-2 [16,17].

Although FISH analysis on CD138-purified plasma cells has greater sensitivity than conventional karyotyping to detect specific genetic alterations [18,19], it has many limitations. Only a limited number of FISH experiments are performed despite the myeloma genetic heterogeneity and the FISH panel offered by clinical laboratories varies greatly in each health care center. Each subsequent FISH study added is associated with a significant burden for clinical laboratories. In addition, FISH is a targeted analysis that does not provide any pangenomic information. Finally, the presence of multiple fluorescent signals and profiles, owing to the genetic complexity of MM, can make analysis and interpretation of results difficult. Therefore, deriving useful and clear conclusions from those patterns presents a significant challenge. This is especially true with *IGH* probes which can be associated with loss of sequences surrounding translocation breakpoints in more than 15% of cases, thus masking a possible *IGH* translocation in a significant number of cases [20,21,22].

Optical genome mapping (OGM) is a highly promising pangenomic method that has the potential to resolve many of the issues and limitations associated with FISH analysis in MM. The cost of an OGM analysis is approximately comparable to a limited myeloma FISH panel (3–4 FISH) and takes 1–2 additional days to complete. It is a straightforward single workflow with an automated analysis pipeline that detects large-scale SVs, both simple and complex, as well as the CNVs that are present across the entire genomes in MM patients. While slightly more time consuming than FISH, it has the potential to provide much greater genomic information and could allow for better prognostication and classification. OGM uses fluorescently labeled (direct label enzyme, DLE) and linearized ultra-high molecular weight genomic DNA with average lengths ranging from 250 kbp to 1 Mb to reconstruct genome maps of SVs and CNVs. To date, the clinical utility of OGM has been validated in other myeloid and lymphoid malignancies [23,24,25,26,27,28] and could replace karyotyping, FISH and chromosomal microarray as a first-tier test in acute leukemias, myelodysplastic neoplasms (MDS) and chronic lymphoid leukemias (CLLs). A pilot study evaluated OGM in the context of extramedullary myeloma, revealing promising results [29]. Thus, it is hypothesized that OGM stands to provide much greater knowledge regarding the genomic complexity and genetic heterogeneity of MM in clinical practice.

The aim of this study was to evaluate the potential of the OGM method to detect clinically relevant aberrations for loci routinely tested in clinical practice by FISH, including all five primary *IGH* translocations, trisomies and monosomies, 17p deletion, chromosome 1 copy number alterations, *MYC* translocations and chromosome 13 aberrations. We also aim to study the potential for OGM to reveal novel SVs and CNVs with potential clinical utility using a more sensitive pangenomic technique. To the best of our knowledge, this is the first study evaluating the feasibility and utility of OGM in clinical practice for MM using CD138-enriched plasma cells.

## 2. Materials and Methods

### 2.1. Sample Selection

From June 2021 to May 2023, 43 newly diagnosed and relapsed myeloma patients, as well as smoldering myeloma and plasma cell leukemia, were prospectively identified from the clinic and enrolled in the study. Approval for this study was obtained from the Maisonneuve-Rosemont Hospital Research Ethics Board and informed consent was obtained for all patients. Bone marrow aspiration (BMA) samples were collected according to institutional protocol for cytogenetic analysis before initiation of chemotherapy. BMA samples were divided into two samples following CD138 immunomagnetic selection only if the enrichment step yielded a minimum of 2.5 million CD138-purified plasma cells. This recovery was mandatory to perform concurrent standard of care (SOC) FISH and OGM analyses. Accordingly, a minimum of one million plasma cells were used for FISH and at least 1.5 million plasma cells were frozen and used for DNA extraction and OGM analysis. Samples with sufficient recovery were then sorted based on the number of bone marrow plasma cells and patients with at least 10% were accepted. Of 43 patients enrolled in the study, 18 patients met the inclusion criteria (i.e., a minimum of 10% plasma cells and a minimum recovery of 2.5 million plasma cells after the enrichment step) and 25 could not be included. Of the 25 patients enrolled but not included in the study, plasma cell counts greater than 10% were not reached for 14 patients, issues during sampling, transportation or freezing were encountered for 5 samples and 5 samples had low CD138 recovery below the established threshold. For these patients, clinical FISH analyses were prioritized and performed without OGM. One patient met the inclusion criteria but was unsuccessful using OGM.

Additional samples, collected with informed consent from two myeloma patients (sample #44 and #45) by the Quebec Leukemia cell bank and with cryopreserved CD138-positive plasma cells, were included in this study to ensure all of the five most frequent *IGH* translocations detected in myeloma would be evaluated in this study and to confirm that these could be adequately detected by OGM. A total of 20 samples were included in this study.

### 2.2. Sample Preparation, CD138 Plasma Cell Purification and Preservation

Four milliliters of BMA were collected in tubes containing 8 mL of RPMI-1640 (#11875-093, Gibco, Grand Island, NY USA) with 500 UI of sodium heparin (#00453811, LEO pharma Inc., Toronto, ON, Canada). Upon reception, 60 µL of DNA stabilizer (Bionano genomics, San Diego, CA, USA) was added to all fresh BMA samples.

Plasma cells were then purified using EasySep™ Human CD138 Positive Selection Kit II (#17887A, Stemcell technologies, Vancouver, BC, Canada) according to the manufacturer’s procedures. Briefly, pelleted BMA samples were washed with RoboSep™ Buffer (#20104, Stemcell technologies) and red blood cells were lysed with 1× EasySep™ Red Blood Cell Lysis Buffer (#20110, Stemcell technologies). Samples were then subject to CD138-positive selection using the semi-automatic Robosep-S™ instrument (STEMCELL Technologies, Vancouver, BC, Canada) using immunomagnetic microbeads coated with anti-CD138 monoclonal antibody. Following CD138 immunomagnetic selection, the positive fraction containing CD138+ cells was resuspended in 2 mL of RoboSep™ Buffer and white blood cell counts were obtained using a Coulter Ac diff 2 counter (Beckman Coulter, Brea, CA, USA). Purity (clinical minimum of >70% plasma cells) of the recovered CD138-positive fraction was confirmed using Wright and Giemsa staining. The remaining plasma cells were mixed once more with DNA stabilizer (Bionano Genomics, San Diego, CA, USA) according to the manufacturer’s recommended ratio of 15 µL per 1 mL of sample, centrifuged to form a dry pellet and frozen at −80 °C until DNA isolation.

Cryopreserved DMSO aliquots of CD138-positive plasma cells from the two MM patients (sample #44 and #45) obtained from the Quebec Leukemia cell bank were thawed at 37 °C for 10 s and washed in RPMI containing 20% fetal bovine serum (Wisent Bioproducts, #080-150). Cells were centrifuged at 1200 rpm for 5 min and pellets were resuspended in 1–2 mL of cold RPMI. Since those cells were cryopreserved, viability was checked for both samples using the methylene blue method. A total of 1.6 and 1.5 million viable plasma cells were used for DNA extraction for sample 44 and 45, respectively.

### 2.3. FISH Analysis

Following plasma cell enrichment, the fractions kept for FISH analysis were subjected to a hypotonic choc using 0.075 M of potassium chloride solution (#609-075-EL, WISENT, Saint-Jean-Baptiste, QC, Canada) for 15 min at 37 °C with 5% CO_2_. Cells were centrifuged at 1200 rpm for 10 min and pellets were resuspended and slow fixed at room temperature for 15 min in a Carnoy solution (1:3 mix of acetic acid and methanol) (#A-0302, #M-3640, APC chemicals, Montreal, QC, Canada) then stored at −20 °C.

Interphasic cells were spread on slides, hybridized with FISH probes and counterstained using 4′,6-diamidino-2-phenylindole (DAPI), according to the manufacturer’s instructions. A total of 5 FISH probes were initially hybridized for each sample based on the algorithm used in our cytogenetic clinical laboratory. Briefly, FISH was conducted in a stepwise sequential manner, according to the number of plasma cells. A TP53 deletion/chromosome 17 centromere FISH probe (Cytocell OGT, LPS 037, Oxford, UK) was used first then followed by an LSI IGH/FGFR3 dual-color dual-fusion probe (Abbott Molecular, 05J74-001, Des Plaines, IL, USA), LSI IGH/MAF dual-color dual-fusion probe (Abbott Molecular, 05J84-004), IGH/MAFB translocation dual-fusion probe (Cytocell OGT, LPH-044) and CKS1B/CDKN2C (P18) amplification/deletion FISH probe (Cytocell OGT, CE-LPH 039). Two hundred nuclei were analyzed for each probe and these results were used in SOC analyses and as a reference for comparison with OGM data. To evaluate concordance, t(11;14) translocations detected by OGM were confirmed by FISH using the LSI IGH/CCND1 dual-color dual-fusion FISH probe (Abbott Molecular). Following SOC analyses, an insufficient quantity of residual plasma cells prevented us from confirming the t(6;14) translocation by FISH for patient 12. Samples 01, 30 and 33 were selected and analyzed using the D5S23/D5S721/CEP9/CEP15 FISH probe (Abbott Molecular, 05N35-020) to confirm HDM classification. The remaining samples without any canonical *IGH* translocation by FISH or OGM, but with recurrent gains of odd-numbered chromosomes as detected by OGM, were classified as HDM. Non-diploid genomes (e.g., triploid or tetraploid genomes) were also classified as HDM if gains of odd chromosomes were observed, without any *IGH* translocation. Non-diploid genomes were suspected when at least three FISH probes showed three or more than three signals for each targeted locus. Cases with rearrangements within the 8q24.21/*MYC* locus were confirmed using the MYC breakapart rearrangement probe (Abbott Molecular, 01N63-020) or the LSI IGH-MYC-CEP8 tri-color dual-fusion probe (Abbott Molecular, 04N10-020).

### 2.4. Optical Genome Mapping

Genomic DNA was extracted from CD138-purified plasma cells from an aliquot of at least 1.5 million cells using the SP DNA extraction kit available. Two different generations of extraction kits were used throughout the study (e.g., generation 1 (#80042, Bionano Genomics) and generation 2 (#80060, Bionano Genomics). Aliquots of 2 million plasma cells were used with a generation 1 extraction kit whereas aliquots of 1.5 million were used for DNA extraction using generation 2. Out of 20 samples processed, 12 were handled with generation 1 whereas 8 were prepared using generation 2.

Cells were processed according to the manufacturer’s instructions for the given generation kit used and without modifications for samples 01–07. Significant issues were encountered with these samples in the first few runs performed with OGM due to the presence of magnetic microbeads in the samples, causing chip clogging and run issues. Most of the samples had to be re-labeled and re-run on the machine at least once, sometimes 2–4 times, in an attempt to achieve quality metrics or enough data for analysis (1500 Gbp). Hence, a microbead removal step was introduced starting at sample 09. After the addition of protease cocktail, vials containing DNA coupled with microbeads were loaded on a magnetic rack for 5 min 3 times using the generation 1 extraction kit and once for 10 min using the generation 2 kit. Once placed on the magnet, beads rapidly agglomerated on the magnetic side of the vial and the supernatant containing the DNA was gently removed and dispensed into a new 1.5 mL tube, taking care not to dislocate the microbead pellet. If required, a second removal step was performed if residual microbeads were still observed (brownish color). It should be noted that loss of DNA was significant during the three 5 min bead removal steps and this step was therefore adjusted to one 10 min step when using the generation 2 kit. Generation 2 kits were found to function better overall for DNA isolation of CD138-positive cells and provided better results (see Section 3). Following bead removal, samples were processed as instructed by the manufacturer, with minor changes. DNA was eluted in a smaller elution buffer volume to obtain concentrated DNA (55 µL; range of 29.4 to 149.67 ng/µL) and DNA was homogenized for 72 h for most samples. A minimum of 620 ng to 750 ng of DNA was used for the labeling reaction. All samples were run on the Saphyr^®^ System (#G199, Bionano genomics) using nanochannel chips (G2.3 (#20366) and G3.3 (#20440), Bionano Genomics) aiming for 1500 Gbp of data for all cases.

### 2.5. OGM Analysis

OGM data were analyzed using the rare variant analysis (RVA) pipeline and visualized using the Bionano Access software v1.7.2. A map rate of at least 70% was expected for all samples, with a coverage of approximately 300× to 400× and 1500 Gbp of data. In cases where non-diploid genomes were suspected (samples 01, 12, 33 and 44) based on FISH results, a de novo analysis was run, in addition to the RVA, using the maximum coverage possible (~250×) to evaluate the potential for ploidy to be detected by the de novo pipeline. Filter settings for RVA were set to default for initial analysis for all cases (minimum number of molecules at 5 and VAF filter 0 and 1), with the following exceptions: SV masking filter and CNV masking filter were set to “All Variants” and “SV in less than or equal to this % of the control db samples” was set to “0” to remove SVs from the normal population.

An additional verification was performed for samples without an *IGH* rearrangement with the intra- and intertranslocation confidence parameter set to “All” to ensure detection of translocations involving telomeric regions, such as the t(4;14) in MM. CNV confidence filters were also adjusted to “All” to look for calls not initially detected by Access using recommended settings, such as the 17p deletion for cases 02 and 42. An in-house cancer gene bed file (Table S1) was developed to filter and detect the clinically relevant abnormalities in MM. Visual inspection of the genome using the “whole genome” function of Access was performed before in-depth analysis to check for possible small aneuploidy clones and small clones with deletion or gain of sequences which might fall below detection limits for a call. Translocations of the *IGH* locus were considered positive when also confirmed by FISH and when breakpoints were detected near the oncogene of interest (*NSD2, CCND1, CCND3, MAF, MAFB*) (up to ~1 Mbp). A cut-off value ˃3 for fractional copy number (fCN) was used to separate 1q gain from 1q amplification.

## 3. Results and Discussion

### 3.1. Patients and Disease Characteristics

From 45 patients participating in this study, 20 patients presented a BMA sample sufficiently enriched in plasma cells and with adequate CD138 recovery to perform both SOC FISH and OGM analyses and were therefore included in this study. Of these 20 patients, 1 presented a smoldering myeloma, 12 patients a newly diagnosed MM, 6 patients a relapsed/refractory MM and 1 patient a secondary plasma cell leukemia. Patients and disease characteristics are described in Table 1. A total of 25 samples were not included in the study (refer to Section 2 for details). Amongst these, 19 were excluded either because they did not meet inclusion criteria or for initial sampling issues unrelated to OGM. Of the six remaining, five were not included because they did not have sufficient CD138 recovery for both FISH and OGM analyses and one sample because of bead removal issues.

The initial number of cells received and the absence of information regarding the number of bone marrow plasma cells in the initial BMA are limitations regularly encountered in clinical laboratories and are the main factors contributing to low recovery of CD138 cells. Clinical labs are routinely faced with bone marrow samples that are not rich enough for a full FISH panel and in these cases OGM could save these samples.

### 3.2. Metrics of OGM Technique on CD138+ Plasma Cells

We were able to obtain sufficient CD138-positive plasma cells to successfully extract DNA and run OGM analysis on all 20 bone marrow samples. The initial DNA extractions performed using the generation 1 kit and without the addition of the bead removal step (samples 01–07), yielded an average of 59.95 ng/µL of DNA (obtained from 1.5–2 million plasma cells) with an average N50 ≥ 150 kbp of 219.38, an average effective coverage of 385.13× and an average map rate of 83.73% (Table S2). With the addition of the bead removal step, we obtained an average DNA concentration of 74.36 ng/µL (obtained from 1.5 million plasma cells), an average N50 ≥ 150 kbp of 267.37, a mean effective coverage of 422.76× and an average map rate of 88.65%. We observed significantly better run metrics overall with the addition of the bead removal step and achieved higher-quality DNA while greatly reducing the need to re-extract or re-label problematic samples. We also noticed an improvement in N50 above 150 kbp (225 versus 290), map rate (84% versus 92%) and effective coverage (398× versus 439×) when switching from generation 1 to generation 2 kits. Overall, these improvements translated into a substantial reduction in scanning time on the instrument.

Sample 45 was not included in the metrics calculation since the total Gbp was significantly below the required 1500 Gbp (1100 Gbp) and this resulted in map rate and coverage lower than our threshold values (<70% and 300×, respectively). However, we included this sample in our study given the presence of an *NSD2*::*IGH*/t(4;14) translocation detected by OGM and confirmed by FISH, which merited further discussion. This sample was also not analyzed in detail beyond the t(4;14) and a global evaluation of SV and CNV calls by OGM.

### 3.3. Overall OGM Results

OGM analysis successfully detected all various SVs (mainly focused on *IGH* translocations and 8q24.21 abnormalities), CNVs (mainly focused on 17p/*TP53* deletion, 1p32/*CDKN2C* deletion and 1q21/*CKS1B* gain/amplification) and aneuploidies (trisomies and monosomies) identified in MM (Table 2, Figures S1 and S2). OGM and FISH data were compared, showing 100% accuracy, sensitivity and specificity for translocations and 92.5% and 95% accuracy for deletions and gains, respectively (Table S3). The lower values obtained for deletions and gains are due to the presence of non-diploid samples in our cohort, which has an impact on the detection of CNVs by OGM (discussed in detail below).

Of the 20 cases, *IGH* translocations were identified in 10 cases and HDM was detected in the other 10 cases, concordant with the expected frequency of these events in MM. In addition to detecting anomalies that are expected with SOC FISH analysis, OGM provided extensive and noteworthy details of the genomes of all 20 patients tested. OGM enabled a more accurate genetic characterization revealing additional prognostic markers in six cases (samples 05, 09, 12, 24, 25, 37), thus representing a 30% increase in prognosis yield at our institution. In addition, OGM permitted better classification for all 10 of our HDM cases. OGM revealed the extent of genomic complexity in myeloma and overall identified a mean of 64 SVs, 63 CNVs and 5 aneuploidy calls per case, not filtering for any specific cancer genes. This is in stark contrast to routine FISH analyses in clinical laboratories that are generally limited to the detection of five classic chromosomal abnormalities. We also observed a difference in the number of SV and CNV calls at diagnosis compared to relapsed cases, with a mean of 60 SVs (range 25–117) and 59 CNVs (range 4–108) with de novo myeloma and an average of 70 SVs (range 30–111) and 68 CNVs (range 31–114) at relapse. It should be noted that these alterations include both clinically pertinent genes/regions as well as those whose clinical relevance is currently unknown. These may include potential false calls, benign calls and alterations that could be of potential interest in the pathogenesis of myeloma. Overall, myeloma genomes were greatly heterogenous with some genomes having very few SVs and CNVs, while others were much more complex with extensive rearrangements and CNVs (Figures S1–S4), consistent with the pilot study looking more specifically at extramedullary myeloma [29].

### 3.4. Successful Detection of Classical Primary Abnormalities in Myeloma by OGM

All five canonical *IGH* translocations, gain of odd-numbered chromosomes and abnormalities of chromosome 13 could be detected by OGM, enabling clear and accurate identification of the primary hit in all patients tested. The OGM findings in comparison to SOC results are shown in Table 2.

**Figure 1:**
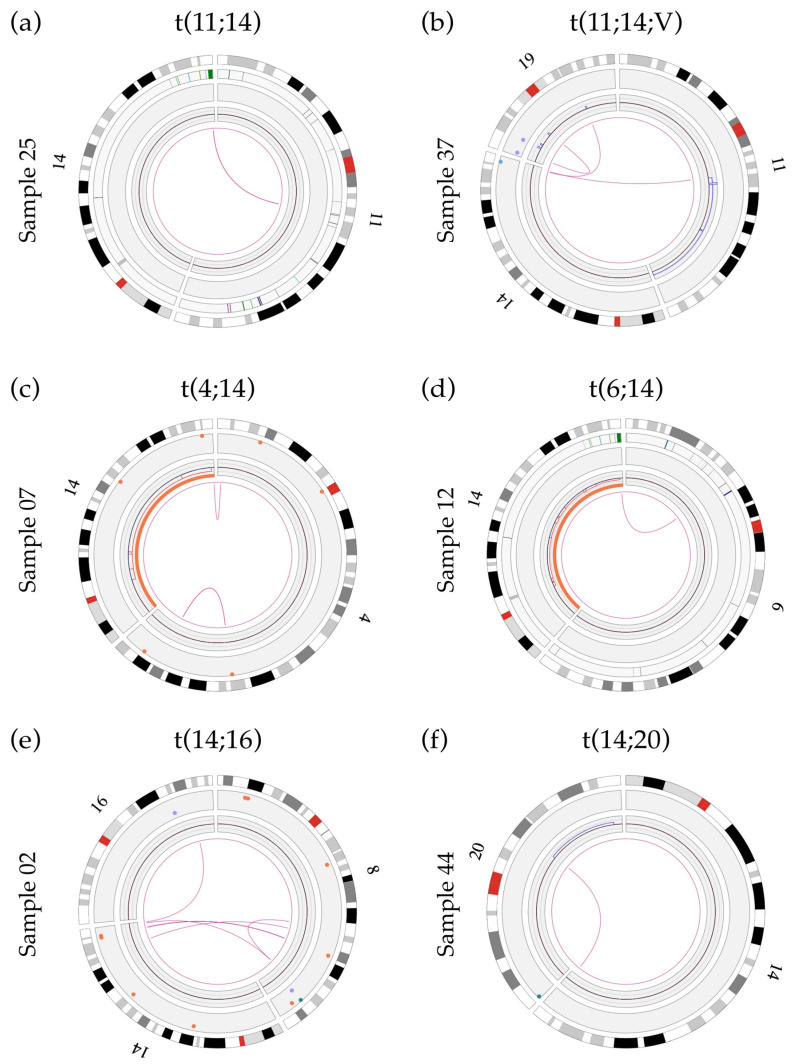
Circos plots showing primary *IGH* translocations: (**a**) Sample 25 showing a two-way t(11;14) translocation involving the *IGH* gene and unknown sequences at chromosomal band 11q13.3. The breakpoint is located between the genes *MYEOV* and *CCND1* and does not involve any previously known gene; (**b**) sample 37 showing a variant t(11;14;19) translocation involving the *IGH* gene and the gene *LINC01488* (between *MYEOV* and *CCND1*) at band 11q13.3. Refer to Section 3.7 for details of t(11;14) breakpoints; (**c**) sample 07 showing a t(4;14) translocation with a rearrangement of the *IGH* gene (14q32) and a disruption of the *NSD2* gene (4p16.3; breakpoint at label 1 901 670 bp); (**d**) sample 12 showing a t(6;14) translocation with a rearrangement of the *IGH* gene and a breakpoint within the *CCND3* gene (6p21.1; break at label 41 969 985 bp); (**e**) sample 02 showing the t(14;16) translocation involving the *IGH* gene and the *WWOX* gene (16q23, break at label 78 739 024 bp); (**f**) sample 44 showing the t(14;20) translocation involving the *IGH* gene and unknown sequences at chromosomal band 20q12. The breakpoint of this translocation (label 40 260 220) is located 427 788 bp in front of the *MAFB* gene (label 40 688 008) and does not involve any previously known gene within this region.

In our cohort, five out of the ten cases with an *IGH* rearrangement were positive for a t(11;14)(q13;q32) translocation by both FISH and OGM analyses (samples 03, 24, 25, 37 and 41), with an estimated frequency of 25% (Table 2). Of these five t(11;14) cases, four showed a classic translocation involving two chromosomes (Figure 1a and Figure S3) while the other exhibited a three-way rearrangement involving a third chromosome partner (sample 37; Figure 1b and Figure S3). We also identified two cases of t(4;14) (sample 07 and 45; 10%) and a single case of t(6;14) (sample 12), t(14;16) (sample 02) and t(14;20) (sample 44) (Figure 1c–f and Figure S3). While a high frequency (~15%) of *IGH* translocations involving loci other than the canonical partners (e.g., *TXNDC5, B2M, JUND, JUN*) is reported in the literature [9,30], such abnormalities were not identified in this small cohort. However, we did uncover two variant translocations (t(14;16;8;8) and t(11;14;19)), both involving at least three translocation partners. These translocations were not characterized in greater detail. Given that novel technologies such as OGM and NGS will reveal and allow characterization of these complex rearrangements, studies are needed to determine if the clinical impact of these variant translocations is the same as that of their classical two-way counterparts, as has been documented in chronic myeloid leukemia with t(9;22;V) [31], and establish whether they respond similarly to treatment.

OGM could identify all *IGH* translocations with stringent settings (confidence filter set to “Recommended”), except for one case (sample 07). However, upon modification to less stringent settings (i.e., inter/intra-chromosomal translocation filters set to “All” instead of “Recommended” and SV masking filter set to “All SVs”) the translocation was called by the Access software v.1.7.2. This is likely due to the low number of labels aligning to the telomeric region of chromosome 4p16, thus leading to a consensus map with a confidence score below the recommended threshold of 0.05 for an SV translocation call. Indeed, translocations involving repetitive sequences, such as telomeric and centromeric translocations, can be missed by the software when using default settings and/or result in lower confidence scores due to difficulty with the alignment of repetitive sequences. Thus, special care must be taken when searching for potential translocations involving these regions, such as the t(4;14) in plasma cells dyscrasias, as these could be overlooked. A need to carefully consider analysis filters has also been reported by other groups studying various hematological cancers [24]. Indeed, a second case of t(4;14) was specifically selected and introduced in this study (sample 45). This second case of t(4;14) was identified without any issues by the software at the default settings. The alignment of the consensus map for the 4p16 region in sample 45 was performed with 14 DLE labels, with a confidence score of 0.28, as opposed to the alignment for sample 07 which was performed using only 7 DLE labels for the same region, with a confidence score of 0.04.

One of the most frequent secondary events observed in cases with an *IGH* translocation was a loss of chromosome 14 which was identified in three *IGH*-positive samples including both samples with t(4;14) and the one with t(6;14) (Table 2; samples 07, 12, 45). The potential for a monosomy 14 in cases with an *IGH* rearrangement provides an excellent example of the potential pitfalls of FISH analysis in MM. In a clinical setting, t(6;14) rearrangement would not typically be included as part of routine SOC analysis as this translocation is not associated with higher-risk disease in current clinical guidelines [12,14]. However, the presence of this translocation can be suspected in a clinical context when using FISH probes specific for t(4;14), t(14;16) and t(14;20) as these would result in three *IGH* signals: a normal *IGH* signal (normal chromosome 14) and a rearranged *IGH* signal split in two (derivative 14 and partner derivative chromosome). The presence of a monosomy 14, in conjunction with a t(6;14), would result in a loss of one of the three FISH signals (Table 2) and would be interpreted as normal, thus masking the presence of this *IGH* translocation and leading to an erroneous clinical interpretation. Lack of proper identification of the t(6;14) translocation prevents us from gaining more knowledge about this genetic rearrangement, which has been included in very few studies since it was first cloned in 2001 [32,33,34]. Having an ability to more readily detect this translocation and other immunoglobulin translocations (*IGH*, *IGL*, *IGK*) may lead to a better understanding of their role and frequency in plasma cell dyscrasias. In this context, OGM is superior to SOC FISH analysis performed in accordance with national and international guidelines.

Of the 20 cases in this cohort, 10 cases with hyperdiploid genomes were identified (samples 01, 05, 06, 09, 18, 30, 33, 38, 42, 43) (Table 2, Figures S2 and S4), consistent with the proportions reported in the literature [35]. Hyperdiploid cases showed a prevalence of gains of chromosome 15 (90%), followed by gains of chromosomes 9 (90%) and 5 (80%) with at least one of these chromosomes expressed in every HDM case (mean gain ranging from 4 to 7 odd chromosomes/case). Additional common gains detected include chromosomes 3, 7, 11 and 19. Of particular interest, in a few cases, OGM revealed not only a single gain but additional copies of chromosomes 5, 15 and 19 with trisomies, tetrasomies and even pentasomies of these chromosomes being detected, irrespective of the ploidy background (Figure S2). In the literature, various definitions have been proposed for diagnosing hyperdiploidy in the context of myeloma, ranging from one or more trisomies of odd chromosomes, with and without *IGH* translocation [18,36], to a broader definition including various quantities of chromosomes in a hyperdiploid state with greater than three copies [37,38]. This discrepancy makes precise identification of HDM more difficult and highlights the need for a consensus definition for accurate identification of this genetic subgroup of myeloma in clinical practice [9]. In this study, any numbers of gains of odd chromosomes classically associated with hyperdiploidy, in the absence of an *IGH* rearrangement, were considered as HDM.

**Figure 2:**
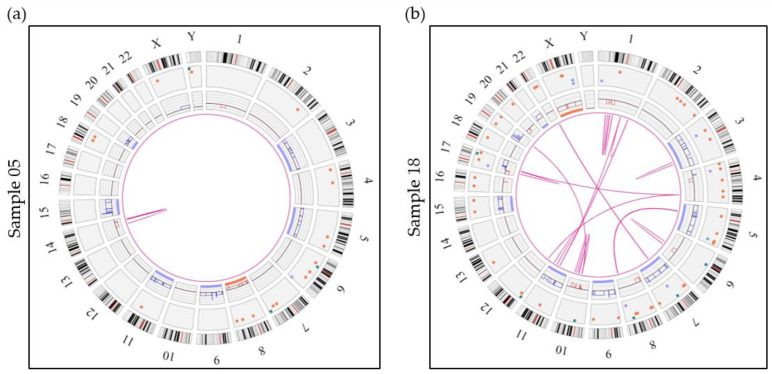
Circos plots showing 2 examples of hyperdiploid genomes. (**a**) Sample 05 represents a hyperdiploid genome (absence of an *IGH* translocation) showing numerous SVs and CNVs including gains of 6 classic odd-numbered chromosomes (3, 5, 9, 11, 15 and 19). Sample 05 also shows an intrachromosomal translocation call by the software, which represents an interstitial deletion of the long arm of chromosome 14. (**b**) Sample 18 shows a much more complex case of hyperdiploidy (absence of an *IGH* rearrangement) with gains of chromosomes 3, 5, 7, 9, 11, 15 and 21 in addition to many more SVs and CNVs. This sample also shows 13 intrachromosomal translocations and 18 interchromosomal translocations. Aneuploidies are represented by blue (gains) and orange (losses) lines in the inner circle and translocations are represented by pink lines in the center. The colored dots in the outer circle represent SVs (deletions, inversions, duplications, insertions).

HDM genomes had an average of 8.4 intrachromosomal translocations, including translocations and interstitial deletions leading to fusion events, and an average of 8.8 interchromosomal translocations. HDM cases showed great variability with regard to their complexity and in the number of associated SVs (Figure 2and Figure S4) with interchromosomal and intrachromosomal translocations ranging from 0–20 and 0–21, respectively. Interestingly, cases with >3 translocations (five cases including two relapse/refractory and three de novo MM; samples 06, 18, 33, 42 and 43) were all positive for loss of the *TP53* gene and showed abnormalities involving the chromosomal band 8q24.21 (rearrangement and gain) but these cases were not necessarily associated with a gain or an amplification of 1q21. Conversely, HDM cases with 0–3 translocations (five de novo cases, samples 01, 05, 09, 30 and 38) were not associated with either *TP53* loss or 8q24.21 abnormalities (Figure S4).

Chromosome 13 abnormalities were detected in 14 of the 20 cases (70%), with 12 cases exhibiting a loss of chromosome 13 (60%) and 2 showing a targeted deletion (10%) (Figures S1 and S2). Loss of chromosome 13 remains the most prevalent event of the two, while targeted deletions are rarer in this cohort, concordant with previous studies [39]. Loss of chromosome 13 was detected in both primary genetic classes, including seven with non-hyperdiploid cases with an *IGH* rearrangement (samples 02, 03, 07, 12, 41, 44, 45) and five with HDM (samples 01, 30, 33, 38, 42). Gene content within the 13q deleted region showed that the *DLEU1* and *DLEU2* genes were included within this region (samples 06, 24), while the *RB1* gene was deleted in only one of the two cases (minimal region of deletion at 13q14.2q14.3). Similar findings were described by Valkama et al., in a CLL cohort [24].

Overall, all primary abnormalities in MM could be detected by OGM irrespective of the genomic context and ploidy levels, for each case. In addition, OGM brings more precision with regard to translocation breakpoints when compared to targeted FISH probes, provides greater characterization of chromosomal rearrangements and allows for a better genetic classification of primary aberrations with respect to future implementation in clinical practice.

### 3.5. Limits Associated with Ploidy Levels in OGM

Currently, OGM is not able to detect whole genome ploidy levels (gain or loss of a whole set of chromosomes) readily and accurately. Regardless of the ploidy status (1n, 3n, 4n etc.), the RVA pipeline normalizes chromosomes to a diploid state (2n). As a result, CNV/SV deletion calls can be erroneously interpreted as monosomies or a single copy of a chromosomal arm/region/gene, while, in reality, these losses may exist on a triploid (or tetraploid) background. Care must be taken when analyzing samples where ploidy may be an issue such as in MM, which is known to have tetraploidy in up to 6% of cases at diagnosis and up to 10% at relapse [40,41]. In this study, four samples had FISH results suggestive of non-diploid genomes (01, 12, 33 and 44; Table 2). While no issues were encountered with regard to the detection of relevant primary translocations or detection of odd-numbered gains, detection and characterization of potential secondary deletion calls of clinical significance present an important challenge in the analysis of these non-diploid samples, as these are associated with higher genetic risk [12,14].

One potential work around would be to use the de novo pipeline instead of, or in addition to, the RVA pipeline, for analysis of MM samples. Odd-numbered ploidy states (e.g., 3n, 5n) have allelic ratios different than the diploid state (variant allele frequency (VAF) of 0.5) and the de novo pipeline could reveal these differential ratios and help identify non-diploid genomes. Indeed, a de novo analysis was additionally run on samples 01, 33 and 44, showing the modified allelic ratios for chromosomes without any SV or CNV alteration at ~250×, which supported the potential for an odd-numbered ploidy state in those samples, concordant with FISH data (Figure S5). However, de novo analysis is only applicable to cases of odd-numbered ploidies, as tetraploid genomes (or any even-numbered ploidy) would likely result in allelic ratios identical to the diploid status and would not be detected, as was the case for sample 12 in our cohort. The presence of multiple SV, CNV and aneuploidy calls can make it difficult to distinguish between a non-diploid genome and a very complex diploid genome and this is further complicated by mosaicism and heterogeneity of subclones, which all contribute to the modification of allelic ratios. In addition, it should be stressed that de novo analysis is a more resource-consuming (hard drive space) and time-consuming analysis compared to RVA, requiring ~24 h for a complete analysis per sample at a coverage of ~250×, which would present significant challenges in the context of a clinical lab.

While consideration of ploidy limits is important in OGM, it does not take away from the overall benefits of OGM and the added precision and pangenomic detail added by OGM when compared to FISH.

### 3.6. Detection of Secondary Abnormalities with OGM

#### 3.6.1. Detection of 17p/TP53 Deletion

**Figure 3:**
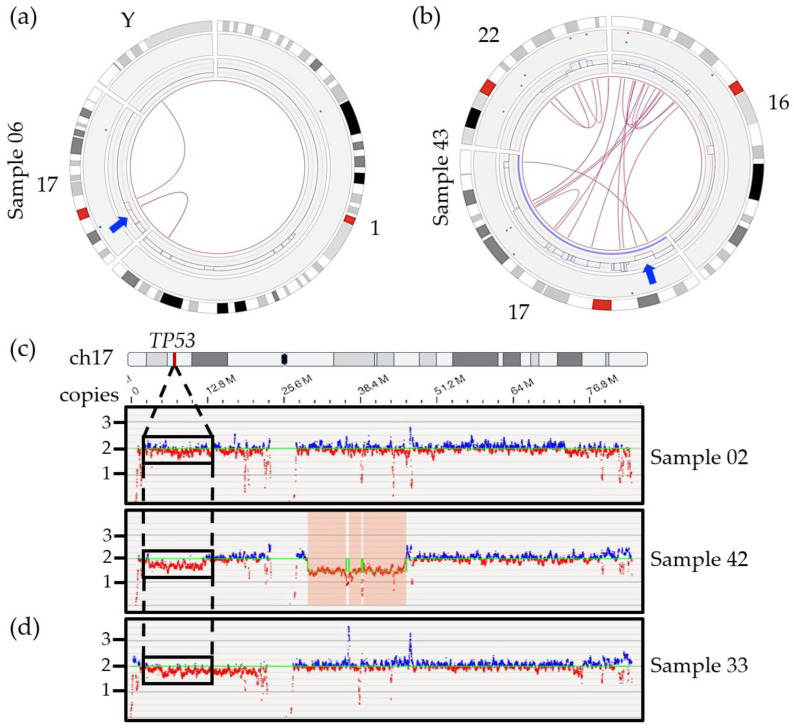
Circos plots of specific chromosomes involved in SVs with chromosome 17 and *TP53* genomic region for two samples. (**a**) Sample 06 revealed two interchromosomal translocations involving the short arm of chromosome 17, chromosome Y and chromosome 1, leading to loss of sequence (red lines) surrounding the breakpoint. The arrow indicates localization of the TP53 gene. (**b**) Sample 43 showing an intrachromosomal translocation call targeting chromosomal bands 17p13.1 and 17q25.5 right next to the deletion region including the *TP53* gene (arrow). Multiple interchromosomal translocations involving chromosomes 16 and 22 also lead to several gains (blue lines) and losses (red lines) on those chromosomes. (**c**) Whole genome view of chromosome 17 for samples 02 (6% by FISH), 42 (45% by FISH) without any deletion call using recommended CNV filter. As opposed to the 17q region which shows both blue and red signals, the absence of blue signals and predominance of red signals correlates with a potential lower copy number for the *TP53* region in those samples. (**d**) Non-diploid sample 33, with 2 and 3 copies of *TP53* by FISH, showing a potential deletion of 17p and potential false positive call.

Among secondary abnormalities, we were able to identify *TP53* deletions in all samples characterized as positive by FISH (Table 2). Four of the six samples were successfully called as a CNV loss by the software (samples 03, 06, 18 and 43) (Figure 3a,b and Figure S1), while the other two samples (02 and 42) were not called but could readily be identified upon visual inspection of the CNV track and whole genome view (Figure 3c). When filter settings were modified beyond the recommended values (CNV confidence set to “All” instead of “Recommended”) the software did call a 9 Mbp deletion of 17p covering *TP53* for one of the two samples (sample 42; confidence score of 0.95, VAF of 0.17 and fCN of 1.66).

The VAF values obtained from OGM were compared to the percentage of positive cells by FISH for samples with *TP53* deletion calls. The percentage of positive cells by FISH corresponds relatively well with twice the VAF, with FISH values ranging from 75% to 98% and VAFs ranging from 0.48 to 0.59 (expected value for a heterozygous call) (Table 2). This is expected since a VAF value for a CNV call is calculated based on the total number of alleles including normal and altered alleles ((fCN-2)/2 = VAF), while FISH analysis only considers the altered allele. Slightly higher VAF values were obtained compared to the expected values based on FISH results. Such a discrepancy is normal given that both techniques are based on, and affected by, different parameters. While OGM uses pooled DNA and provides an average allelic frequency based on the total number of cells, FISH is impacted by other factors including hybridization issues and human interpretation of results, which can impact the calculation of positive cells. We also found that fCN values specifically for the *TP53* gene ranged from 0.8 to 1.04, correlating with one residual copy of the gene, as confirmed by FISH. Overall, OGM could correctly estimate the proportion of cells harboring the *TP53* deletion for all samples with a deletion call.

In addition, OGM was able to provide significant detail on the larger genomic context for the *TP53*/17p deletion and showed a trend toward more SV and CNV calls in all positive cases compared to samples without 17p/*TP53* deletion (SV: 80 vs. 57; CNV: 81 vs. 55). We could also show that *TP53* deletions, in this cohort, did not result from small, targeted deletions as has been reported in CLL and acute myeloid leukemia (AML) [42,43,44,45] but rather from large 17p deletions (Figure 3a,b and Figures S3 and S4). The deletions ranged from 7 Mbp to 22 Mbp with an average deletion size of 16 Mb (size of 17p = 22.6 Mbp) and minimally included the chromosomal bands 17p13.1 to 17p12 in the five patients with an OGM call (samples 03, 06, 18, 42, 43; strict and relaxed filters). OGM further revealed that these deletions were the result of unbalanced SVs, most often interchromosomal translocations involving one or more chromosomal regions, which lead to gains and losses of flanking regions (Figure 3a,b and Figures S3 and S4). Of note, we could observe the presence of an 8p deletion in five out the six patients harboring a 17p/*TP53* deletion while only one case with an 8p deletion was identified within the fourteen other cases without any CNV call in this 17p region (Figure S1).

The four samples that were detected by the Access software using the recommended filters had relatively high deletion frequencies by FISH (75–98%) while the two discordant cases had a proportion of positive cells that was significantly lower. The lack of a call by Access for sample 02 may be partly explained by the limit of detection of the software. Current studies performed in AML, MDS and CLL suggested that CNV losses, such as the 17p deletion in myeloma, could be missed if detected at a VAF lower than 5–16% [24,28,46,47,48,49]. Indeed, sample 02 presented only 6% positive cells by FISH (Table 2). The absence of a CNV call with recommended filters for sample 42 is unlikely to be due to low mosaicism given the relatively high percentage of positive cells (45%). The fact that only one fluorescent DLE label falls within *TP53* itself, resulting in poor coverage for the gene, is also unlikely to be a contributing factor for the lack of a call in this case since a visual inspection of this region also suggests the presence of a large-scale deletion impacting a large portion of the short arm of chromosome 17 (Figure 3c). While the lack of a CNV or SV call presents a potentially important limit of the RVA pipeline for the *TP53* deletion, we did find that we had sufficient visual evidence to be fairly confident in the presence of a deletion for both samples 02 and 42. Visual inspection of the short arm of chromosome 17 using the whole genome view and checking for calls using less stringent CNV filters are highly recommended steps when analyzing a hematopoietic disease classically known to have a potential *TP53* deletion, owing to its prognostic impact. In cases of uncertainty, FISH or another method, such single nucleotide polymorphism array (SNP-A) or NGS, should be performed for confirmation.

The potential for false positives with *TP53* may present a more important challenge in OGM. Figure 3d provides an example of the whole genome view for sample 33, which is non-diploid. Visual inspection of the 17p region suggests a potential 17p/*TP53* deletion, however, FISH did not find any clinically significant deletion, with two and three copies of the gene observed (Table 2). Given the clinical definition of a *TP53* deletion (a single copy remaining), this would not be considered a true clinically relevant deletion. For now, a secondary method of confirmation may be necessary in cases with *TP53* deletion calls to ensure that they are not the result of ploidy issues. Further studies are needed to better understand the limits of detection of OGM in cases of deletion to minimize the possibility of false negative and false positive calls.

The 17p/*TP53* deletion is the most important independent prognostic marker accepted in several approved risk scores routinely used in clinical settings [50]. Deletion of 17p/*TP53* defines a high-risk category of patients, associated with advanced disease state and with chemotherapy resistance [51], highlighting the need for proper and accurate identification. As discussed above, detection of 17p/*TP53* can be challenging with OGM especially in cases with a lower fraction of positive cancer cells. However, clone size is still a matter of debate in myeloma, with different cut-off values proposed by myeloma experts (10%, 20%, 55% and 60%) [52,53]. Despite the literature demonstrating that as the clone size increases the overall survival decreases, no consensus and no cut-off value are yet accepted or incorporated into current diagnosis and treatment algorithms (https://www.msmart.org/mm-treatment-guidelines (accessed on 16 August 2023)) [12,14,54,55]. Regardless of the minimum percentage of positive cells required, accurate identification of this deletion is of crucial importance as it guides treatment decisions and allows for discrimination of patients with higher risk of progression. *TP53* calls (or lack thereof) along with their associated VAF values will need to be carefully evaluated before routine implementation of OGM in clinics, as false positive or false negative calls will be of major clinical consequence.

#### 3.6.2. Chromosome 1 Abnormalities

**Figure 4:**
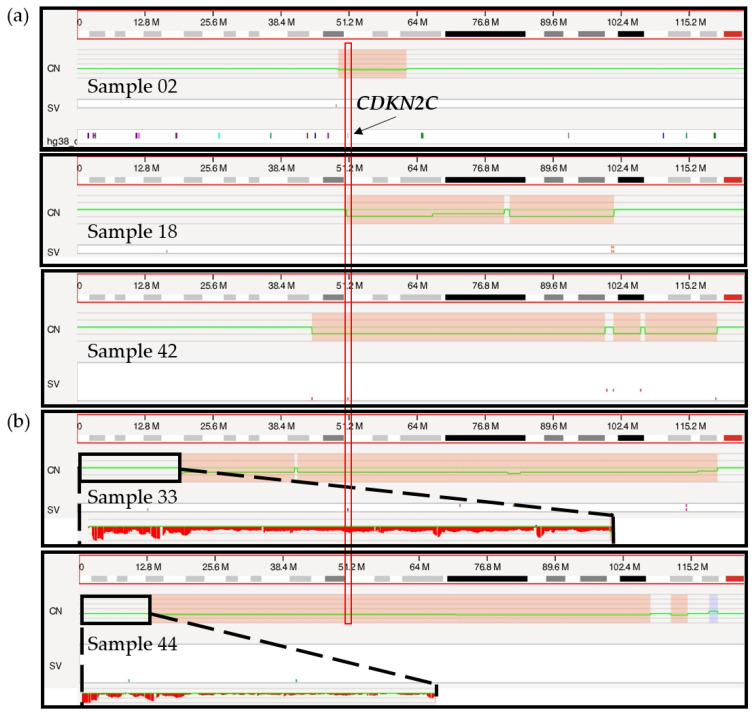
Genome browser view in Access of the short arm of chromosome 1 from the telomere to the centromere. (**a**) Targeted 1p/*CDKN2C* deletion calls for samples 02, 18 and 42 are indicated in red boxes on the copy number (CN) track with a concurrent change in the copy number (green line). The vertical red rectangle indicates the localization of the *CDKN2C* gene, at chromosomal band 1p32.3. (**b**) Larger 1p/*CDKN2C* deletion calls for sample 33 and 44 marked in red on the CN track. Sequences located on the telomeric end of chromosome 1p are zoomed in (black rectangle), showing a potential loss of sequences by visual inspection.

Deletions in the short arm of chromosome 1 targeting the *CDKN2C* gene located at chromosomal band 1p32 were correctly called by OGM in all three samples with concordant FISH results (02, 18, 42; Figure 4a). The size of the deleted region varied from 12 Mbp to 73 Mbp, minimally targeting chromosomal bands 1p32.3 to 1p31.3. OGM called an additional 1p/*CDKN2C* deletion in two other cases (samples 33 and 44) (Figure 4b), with a fractional copy number of 1.37 and 1.72 suggesting the presence of possibly one to two copies of 1p (sample 33: VAF 0.32; sample 44: VAF 0.14). As discussed above, FISH analysis supports the presence of non-diploid genomes in these two cases, with three or more copies of most loci tested including the 1q21 region *(CKS1B*). The only exception was with the probe specific for *CDKN2C*, which showed two copies of the gene in all cells for sample 33 and two copies of the gene in 43.9% of cells for sample 44. Taken together, these FISH results point to the likely presence of at least three copies of chromosome 1 sequences, harboring either a targeted deletion of 1p on the third chromosome or an additional copy of the long arm of chromosome 1 (1q), leading to a different allelic ratio between 1p and 1q and, therefore, to a deletion call by the RVA pipeline. However, while a third copy of 1p may be missing in those patients, two copies remain, and as with the *TP53* deletion for sample 33, these calls should not be considered clinically significant.

It is interesting to note that the 1p deletion calls observed for samples 33 and 44 are of a noticeably larger size (>100 Mbp), involving the whole short arm of chromosome 1 in both cases (Figure 4b), compared to the more targeted calls of the three confirmed cases (12–73 Mbp). However, a larger sample size is necessary to better understand the significance of this distinction and determine if this could be an accurate and repeatable method to infer a potential erroneous 1p deletion call.

A second deleted region on chromosome 1p was revealed by OGM at chromosomal band 1p12 in samples 05, 07, 09 and 42 (Figure S6). However, this deletion was not otherwise confirmed by an orthogonal method. Nonetheless, given the recurrence of this targeted deletion in multiple samples in this cohort and the fact that it is a well-described deletion region in myeloma patients [56,57], we are confident that this is a true deletion call. In addition, all four samples with the 1p12 deletion were found to have a diploid genome by FISH and therefore we do not expect ploidy to have an impact on this call. This region of deletion minimally included chromosomal bands 1p13.2 to 1p11.2, with size ranging from 4 Mbp up to 73 Mbp. This region of deletion, previously described by Walker et al., in 2010, was shown to include the *TENT5C* (previously known as *FAM46C*) gene and was reported as a separate deletion from 1p32/*CDKN2C* [56,57]. Interestingly, in our cohort, the gene *NRAS* was also identified within this recurrent region of deletion (Figure S6). This is of particular interest as both *TENT5C* and *NRAS* are known to be mutated in 9.4% and 17.5% of myeloma cases, respectively [30], and activating mutations of the *NRAS* gene were shown to significantly reduce myeloma sensitivity to single-agent bortezomib but not high-dose dexamethasone [58,59]. It is interesting to consider if a deletion of *NRAS* could have the opposing effect and increase sensitivity to bortezomib in these patients. A recent study showed that patients with an isolated 1p deletion, which included chromosomal regions 1p32p12, had a 40-fold reduction in their rate of progression when treated with lenalidomide maintenance following autologous stem cell transplant [60] thus highlighting the importance of identifying both 1p deletion regions in myeloma.

Gains of 1q (three copies of the *CKS1B* gene) were correctly called by OGM in all cases positive by FISH (samples 02, 06, 24, 30, 37, 38 and 41) (Table 2, Figure S7). OGM showed an fCN value ranging from 2.32 to 3.05, corresponding with three copies of *CSK1B,* as confirmed by FISH, in all seven cases. According to OGM, the size of the 1q gain was relatively consistent in all samples (size from 100 to 103 Mbp), covering a large part of the long arm of chromosome 1 (1q: 121 Mbp). Moreover, as with 17p deletions, 1q gains were more often the consequence of SVs (five of the seven cases), usually interchromosomal translocations (Figures S3 and S4), resulting in gains and/or losses of sequences near the breakpoints. This was also observed in the pilot study [29]. Overall, SV and CNV calls were not increased in cases with a 1q gain compared to cases with two normal copies of 1q (SV: 62 vs. 59; CNV: 55 vs. 62).

**Figure 5:**
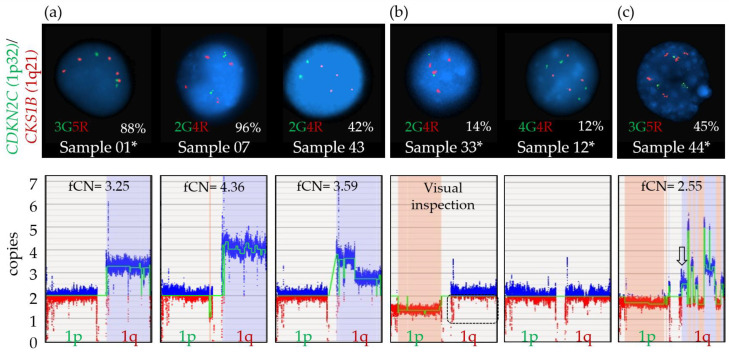
Amplification 1q calls by FISH and OGM. (**a**) Amplification of 1q is defined as detection of at least four copies of the *CKS1B* probe by FISH. Upper panel: Representative interphasic nuclei of each case showing at least four copies by FISH for this specific locus (red signal), with various clone sizes as indicated by percentage of positive cells (sample 01, 07, 43). Green signals represent the 1p32 locus on chromosome 1. Lower panel: Amplification call by OGM, as defined by a fractional copy number ≥ 3 for each sample. (**b**) Upper panel: Representative interphasic nuclei of samples showing four *CKS1B* signals by FISH (red signal), with clone size below sensitivity of the OGM method (sample 33, 12). Lower panel: Genome view of the short arm (1p) and long arm (1q) of chromosome 1 shows a decreased intensity of red markers (dotted rectangle) compared to blue markers for sample 33 but such variation cannot be identified for sample 12. (**c**) Upper panel: Representative interphasic nuclei of sample 44 showing a plasma cell with five *CKS1B* signals by FISH (red signal). Lower panel: As visualized from the genome view of the long arm (1q) of chromosome 1, various fCN values are detected, ranging from 1.65 to 5, with an fCN specific for the *CSK1B* region of 2.55 (arrow). * Samples with a non-diploid genome. In the lower panels, blue boxes represent gains and red boxes represent losses.

Amplification of 1q (>3 copies) could be readily identified and distinguished from 1q gain by OGM in three (01, 07, 43) of the six samples with amplification confirmed by FISH (Table 2). Generally, an fCN between 2 and 3 should represent cases with a population of cells with three copies of 1q and an fCN above 3 would indicate four or more copies of 1q. Samples 07, 43 (diploid) and 01 (non-diploid) all had an fCN value above 3 (4.36, 3.59 and 3.25), consistent with the definition of amplification (more than three copies of *CKS1B* by FISH) and with FISH results (Table 2, Figure 5). All three remaining samples were non-diploid genomes based on FISH (12, 33 and 44). Despite some evidence pointing to a potential increased copy number of 1q in samples 33 and 44 (Figure 5), no clear separation could be made between a gain and an amplification of 1q21/*CKS1B* for these as ploidy interferes with the identification of the real copy number for the *CKS1B* gene.

FISH percentages and fCN values were found nearly equivalent for sample 07 with four copies of *CKS1B* in 96% and an fCN of 4.07 as well as for sample 43 which showed four copies of *CKS1B* in 42% and an fCN of 3.59, consistent with an amplification using both parameters. For the remaining samples (01, 33, 12 and 44), the fCN values were not exactly comparable to FISH data (Table 2), in large part due to the non-diploid genomes suspected by FISH for all four and mosaicism within plasma cells which impact fCN as well as VAF values. In theory, as sample 01 and 44 harbored non-diploid genomes with at least 3n, fCN values need to be adjusted with +1 to obtain a more appropriate estimate of the copy number within this region. Therefore, concordance between FISH and fCN values is not possible in non-diploid cases. As gains and amplifications of 1q must be differentiated for prognostication purposes [61], future studies will be needed to establish a clear delineation between these two types of CNVs using OGM.

However, apart from non-diploid cases which represented a limitation of OGM, the novel pangenomic method was able to detect all the classic secondary abnormalities in all diploid samples, including gains and amplifications as well as deletions.

#### 3.6.3. 8q24.21/MYC Abnormalities

Various anomalies of chromosomal band 8q24.21 involving the *MYC* locus were identified in 11 of the 20 samples in this cohort (55%). Nine of these samples (02, 07, 09, 33, 18, 30, 06, 24, 37) showed either inter- or intrachromosomal translocations involving the chromosomal band 8q24.21 with either immunoglobulin loci (i.e., 14q32.33 (*IGH*) and 22q11.22 (*IGL*)), or various other partners (i.e., 15q21.2 (*MIR4713HG*), 4q13.3 (*RUFY3*), 5q11.2 (*ANKRD55*), 5q33.3 (*TIMD4*), 8q21.3 (*CNBD1* and *MMP16*), 8q22.3 (*RIMS2*), etc.) by OGM. Only sample 07 resulted in a simple two-way interchromosomal translocation. These were confirmed by FISH, using the MYC breakapart probe, in five samples (02, 07, 33, 06 and 24) and one sample could not be confirmed due to lack of material (sample 37). Most of these translocations were the result of more complex rearrangements with combinations of inversions, deletions, insertions and/or multi-way translocations involving other chromosomes or other regions of the long arm of chromosome 8 (Figure S8). Interestingly, FISH analysis was normal for samples 09, 18 and 30, likely due to the fact that these SVs were the result of small insertions and/or small inversions of sequences within the PVT1/MYC region and did not lead to signal separation (Figure S8). Two of the eleven cases with an abnormality of the *MYC* locus showed a deletion instead of a translocation in this region, completely encompassing or partially deleting the *PVT1* gene (samples 05 and 38; Figure S8). A biallelic loss was observed in sample 05, resulting from a combination of a monosomy 8 and a targeted deletion of 480 Kbp in size completely encompassing the *PVT1* gene (fCN: 0.07–0.19; VAF 0.83). A similar monoallelic and targeted deletion of 2.4 Mbp was also observed in sample 38, with a complete deletion of the *MYC* gene and partial deletion of the 5′ region of the *PVT1* gene. Rearrangements involving the *MYC* locus, detected by FISH, are associated with an adverse prognosis in patients with MM [15]. Further investigations are warranted to evaluate the prognostic impact of these rearrangements and other abnormalities involving 8q24.21 detected by more sensitive methods, including OGM.

### 3.7. Significant Variability Found in Translocation Breakpoints

**Figure 6:**
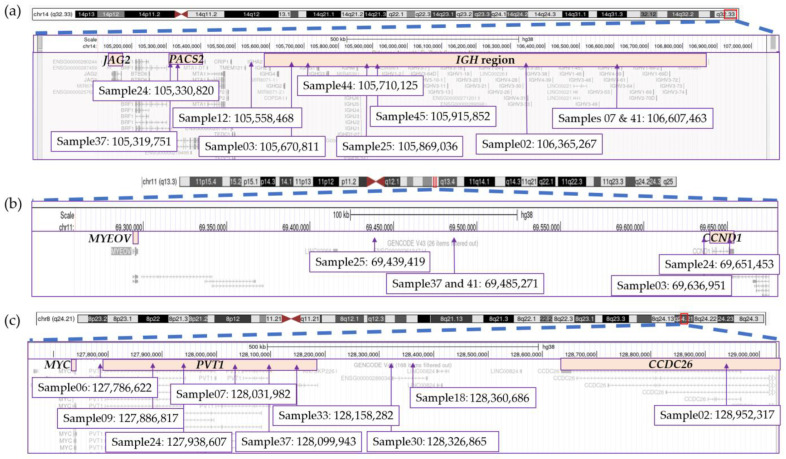
Breakpoints for structural variations involving the (**a**) *IGH* region on chromosome 14, (**b**) *CCND1* region on chromosome 11 and (**c**) *MYC* region on chromosome 8. This figure was modified from screenshots of the hg38 genome taken from the UCSC Genome Browser (http://genome.ucsc.edu (accessed on 9 June 2023)) [62].

In addition to identifying all recurrent canonical rearrangements, OGM provided clarity and detail with regard to the location of breakpoints involved in translocations (Figure 6). Overall, we found significant variability in the breakpoints and genes involved in the *IGH* rearrangements as well as those involving the 8q24.21/*MYC* locus. A distance of 1.28 Mbp was identified between the two furthest breakpoints within the *IGH* region located at chromosomal band 14q32.33, as determined by OGM (Figure 6a). Six of the ten rearrangements had breakpoints within the *IGH* locus, with four samples having breakpoints involving the VDJ region and two falling within the constant region (Figure 6a). The remaining three translocations had breakpoints centromeric to *IGH* with two of these having breakpoints involving the *PACS2* gene. Similarly, breakpoints in chromosome 11 from t(11;14)-positive rearrangements covered a distance of more than 200 Kbp and involved several genes and regions outside of the *CCND1* gene (Figure 6b). Only sample 24 had a breakpoint estimated to disrupt the *CCND1* while sample 03 showed a breakpoint in proximity to but outside of the *CCND1* gene.

The *PVT1* gene at 8q24.21 represents the main target of rearrangements and structural variants (deletions, insertions, inversions) in this region, not the *MYC* gene, followed by the *CCDC26* gene (Figure 6c). No sample was found to have a breakpoint directly falling within the *MYC* gene. Breakpoints were spread across the region, spanning a large distance of approximately 1.2 Mbp between the two furthest breakpoints (Figure 6c).

The results presented herein are consistent with previous studies that have identified large distances between translocation breakpoints on chromosomes involved in *IGH* and *MYC* rearrangements [63,64,65]. Even in cases with breakpoints >1 Mbp apart, these studies detected overexpression of the relevant oncogenes. The impact of this variability in breakpoints needs to be further explored as the involvement of different genes within an identical translocation could influence response to treatment in MM patients and have further impact on survival and progression. A recent study looking specifically at breakpoints in or near the *NSD2* gene (alias *MMSET*), involved in t(4;14) translocation, determined that the location of the breakpoints had a significant impact on survival of patients [66]. Breakpoints located further inside the coding region of *NSD2* resulted in lower survival curves compared to breakpoints outside of the coding region. However, t(4;14) currently represents one of the few recurrent translocations in myeloma known to generate a chimeric fusion transcript [9,66]. Breakpoints occurring within oncogenes were also observed in a few patients in our cohort, disrupting the *CCND3*/t(6;14), *CCND1*/t(11;14) and *WWOX*/t(14;16) genes, as well as the *NSD2*/t(4;14) gene.

Given that OGM is not a sequencing technology and relies on the successful integration of fluorescent DLE labels at specific regions in the genome, it is important to note that the exact base pair location of the breakpoints cannot be determined. The Bionano Genomics Solve Theory of Operation for Structural Variant Calling reports a typical uncertainty of approximately 3 kbp in the location of the breakpoint (with the 90th percentile at ~11 kbp). An alternative method, such as whole genome sequencing, would be appropriate to confirm the exact breakpoint locations, though that is beyond the scope of this study. Nevertheless, the OGM technology does allow increased specificity in the identification of breakpoints as compared to FISH and highlights the variability of breakpoints in these recurrent rearrangements.

## 4. Conclusions

OGM is a straightforward technique with minimal hands-on time that can be completed (extraction to analysis) in 5 to 7 days. With its ability to provide a pangenomic view of the malignant plasma cell genome and the breadth and depth of information on abnormalities, OGM stands to greatly improve our knowledge and understanding of myeloma genetics. OGM can better characterize currently known abnormalities, clarify the breakpoints for recurrent translocations, provide greater genomic context for SVs and CNVs and bring new abnormalities to the foreground. Overall, OGM provided additional prognostic information for 6 of the 20 evaluated cases, representing a 30% increase in prognosis yield. Care should be taken when analyzing the 17p13.1/*TP53* and *CKS1B*/1q21 chromosomal regions as calls in these regions could be impacted by ploidy. In cases of uncertainty, FISH should be used. Despite some limitations, our results support the usefulness of OGM in the context of a clinical laboratory and its ability to provide a thorough genetic profiling of myeloma patients, offering the possibility of much more accurate prognostic classifications based on genetic risk.

Myeloma is a very heterogeneous disease, from both a clinical perspective and a genetic view. Even within a single genetic category and subclass, patients with identical translocations or CNVs progress differently, with highly heterogeneous survival. This reflects the need to improve standard laboratory practices to include a global vision of myeloma genomes, not limited to a few targeted abnormalities. The introduction of new genome-wide methods provides a deeper understanding of this disease and enables the identification of novel genomic, prognostic and predictive markers that will improve patient management. As new technologies advance, up to date clinical guidelines that incorporate these new and complex findings will be urgently needed to guide clinical practice.

## Figures and Tables

**Table 1:** Patients and disease characteristics.

Samples	Age *	Disease	Prior Lines of Therapy	Isotype	BM Plasma Cells/cPC (%)	R-ISS	HD-MEL	Interval from HD-MEL to Sample (Months)	Other Characteristics
1	61	NDMM		IgG kappa	26/0	1	No		
2	59	RRMM	3	IgA kappa	60/0	NA	Yes	7	
3	56	RRMM	1	FLC kappa	28/-	NA	Yes	19	
5	67	NDMM		IgG kappa	70/0	2	No		
6	72	RRMM	6	IgA kappa	36/0	3	No		Radiation therapy for prostate cancer 11 years before sample collection
7	68	sPCL	4	IgG lambda	94/13	3	Yes	48	
9	58	NDMM		IgG kappa	19/0	1	No		
12	82	SMM		IgG lambda	10/0	-	No		Recent diagnosis of MDS at sample collection
18	71	RRMM	6	IgG kappa	35/0	2	Yes	81	ASCT tandem
24	82	NDMM		FLC lambda	25/0	2	No		
25	72	NDMM		IgG kappa	60/-	2	No		
30	65	NDMM		IgG lambda	65/-	2	No		
33	62	NDMM		FLC lambda	80/1	3	No		
37	76	NDMM		FLC kappa	25/0	2	No		
38	69	NDMM		IgG kappa	97/0	3	No		
41	84	RRMM	4	IgA kappa	33/-	NA	No		
42	79	NDMM		IgG kappa	65/0	NA	No		
43	83	NDMM		IgA kappa	48/0	3	No		
44	88	NDMM		IgG kappa	14/0	2	No		
45	59	RRMM	1	IgA kappa	44/3	NA	Yes	6	

Abbreviations: ASCT: autologous stem cell transplantation; cPC: circulating plasma cells; NDMM: newly diagnosed multiple myeloma; FLC: free light chain; HD-MEL: high-dose melphalan; NA: not available; MDS: myelodysplastic neoplasm; R-ISS: revised International Staging System; RRMM: relapsed/refractory multiple myeloma; SMM: smoldering myeloma; sPCL: secondary plasma cell leukemia. * At time of study participation.

**Table 2:** Comparison of standard of care FISH results and OGM.

Samples	FISH (% of Abnormal Cells)	OGM (fCN/VAF)	Concordance
1 (non-diploid)	*TP53*/D17Z1 (4 × 81%)	Gain of chromosome 17	Yes
	*FGFR3* (3 × 90%)	Gain of chromosomes 5, 6, 7, 9, 15, 19	Ploidy *
	*IGH* (3 × 45%, 4 × 46%)	Gain of chromosome 14	Yes
	*MAFB* (3 × 94%)		Ploidy *
	1p32/*CDKN2C* (3 × 88%)		Ploidy *
	1q21/*CKS1B* amplification (5 × 88%)	1q21/*CKS1B* amplification (3.25/0.63)	Yes
2	Deletion 17p/*TP53* (1 × 6%)	Deletion 17p/*TP53* (visual inspection)	Yes
	t(14;16) (2F 98%)	t(14;16) (0.48)	Yes
	Deletion 1p32/*CDKN2C* (1 × 22%)	Deletion 1p32/*CDKN2C* (1.73/0.135)	Yes
	1q21/*CKS1B* gain (3 × 71%)	1q21/*CKS1B* gain (2.72/0.36)	Yes
3	Deletion 17p/*TP53* (1 × 95%)	Deletion 17p/*TP53* (0.91/0.54)	Yes
	t(11;14) (2F 77%, 3F 17%)	t(11;14) (0.55)	Yes
5	Normal		Yes
		Gain of chromosomes 3, 5, 9, 11, 15, 19	N/A
6	Deletion 17p/*TP53* (1 × 75%)	Deletion 17p/*TP53* (0.81/0.59)	Yes
		Gain of chromosomes 3, 7, 11, 15	N/A
	1q21/*CKS1B* gain (3 × 70%)	1q21/*CKS1B* gain (2.86/0.43)	Yes
7	t(4;14) (1F 97%)	t(4;14) (0.51)	Yes
	1q21/*CKS1B* amplification (4 × 96%)	1q21/*CKS1B* amplification (4.36/1.18)	Yes
9	*IGH* (1 × 6%)	Gain of chromosomes 3, 5, 9, 11, 15, 19, 21	No
12 (non-diploid)	*TP53*/D17Z1 (4 × 14%)	t(6;14) (0.52)	Ploidy *
	*FGFR3* (4 × 16%)		Ploidy *
	*IGH* (3x 18%, 4 × 13%)		Yes
	*MAF* (4 × 14%)		Ploidy *
	*MAFB* (4 × 10%)		Ploidy *
	1p32/*CDKN2C* (4 × 12%)		Ploidy *
	1q21/*CKS1B* amplification (4 × 12%)	1q amplification not detected	Ploidy *
18	Deletion 17p/*TP53* (1 × 98%)	Deletion 17p/*TP53* (1.02/0.49)	Yes
	Deletion 1p32/*CDKN2C* (1 × 96%)	Deletion 1p32/*CDKN2C* (1.05/0.47)	Yes
		Gain of chromosomes 3, 5, 7, 9, 11, 15, 21	N/A
24	t(11;14) (2F 87%)	t(11;14) (0.24)	Yes
	1q21/*CKS1B* gain (3 × 95%)	1q21/*CKS1B* gain (2.92/0.46)	Yes
25	t(11;14) (2F 97%)	t(11;14) (0.69)	Yes
30		Gain of chromosomes 3, 5, 7, 15, 18, 19	N/A
	1q21/CKS1B gain (3 × 60%)	1q21/*CKS1B* gain (2.41/0.20)	Yes
33 (non-diploid)	*TP53*/D17Z1 (2 × 17.5%, 3 × 70%)	Deletion 1p32/*CDKN2C* (1.37/0.32)	Ploidy *
	*FGFR3* (3 × 64%)	Gain of chromosomes 3, 5, 7, 9, 15	Ploidy *
	*MAFB* (3 × 91%)		Ploidy *
	1q21/*CKS1B* (3 × 81%, 4 × 14%)	1q21/*CKS1B* gain by visual inspection	Yes
37	t(11;14) (1F 89%)	t(11;14) (0.45)	Yes
	1q21/*CKS1B* gain (3 × 49%)	1q21/*CKS1B* gain (2.32/0.16)	Yes
38		Gain of chromosomes 9, 11, 15, 19	N/A
	1q21/*CKS1B* gain (3 × 91%)	1q21/*CKS1B* gain (3.05/0.53)	Yes
41	t(11;14) (2F 96%)	t(11;14) (0.25)	Yes
	1q21/*CKS1B* gain (3 × 95%)	1q21/*CKS1B* gain (2.99/0.49)	Yes
42		Gain of chromosomes 5, 9, 11, 15, 18, 19	N/A
	Deletion 17p/*TP53* (1 × 45%)	Deletion 17p/*TP53* by visual inspection	Yes
	Deletion 1p32/*CDKN2C* (1 × 78%, 0 × 12%)	Deletion 1p32 CNV (1.08/0.46), SV targeted deletion *CDKN2C* (0.9/0.22)	Yes
43		Gain of chromosomes 3, 5, 7, 9, 11, 17, 18, 19	N/A
	Deletion 17p/*TP53* (1 × 97%%)	Deletion 17p/*TP53* (1.04/0.48)	Yes
	1q21/*CKS1B* amplification (3 × 29%, 4 × 42%; 5 × 19%)	1q21/*CKS1B* amplification (3.59/0.8)	Yes
44 (non-diploid)	*TP53*/D17Z1 (3 × 5%, 4 × 17%, 5 × 41%)	Gain of chromosome 17	Yes
	*FGFR3* (3 × 59%)		Ploidy *
	*MAF* (3 × 35%)		Ploidy *
	t(14;20) (2F 66%, 3F 43%)	t(14;20) (0.28)	Yes
	1p32/*CDKN2C* (3 × 50,4%)	Deletion 1p32/*CDKN2C* (1.72/0.14)	Ploidy *
	1q21/*CKS1B* amplification (3 × 4%, 4 × 21%, 5 × 45%)	1q21/*CKS1B* gain (2.55/0.68)	Ploidy *
45 (non-diploid)	*TP53*/D17Z1 (3 × 67%)	Deletion 1p32/*CDKN2C* (1.42/0.29)	Ploidy *
	t(4;14) (2F: 8%; 3F: 85%)	t(4;14) (0.28)	Yes
	1q21/*CKS1B* amplification (5 × 4%, 6 × 28%, 7 × 49%, 8 × 12%)	1q21/*CSK1B* amplification (4.15/1.08)	Ploidy *

FISH results, showing the copy number and the percentage of positive cells, were compared to the fractional copy number (fCN) and variant allele frequency (VAF) values obtained by OGM for the 17p/*TP53* deletion, various *IGH* translocations and chromosome 1 abnormalities. Probe targeting *TP53*/D17Z1 loci, t(4;14), t(14;16) and t(14;20) dual-color dual-fusion probes and probe for detection of 1p32/*CDKN2C* deletion and 1q21/*CKS1B* amplification were used for every sample. For patients with *IGH* translocations, the number of fusion signals is reported. If not otherwise specified, results were consistent with the normal FISH signal pattern expected with the specific probe. Samples with non-diploid genomes are indicated. Ploidy * refers to cases with non-diploid genomes where concordance could not be readily evaluated. N/A: not assessed by FISH.

## Data Availability

To our knowledge, no online repository currently exists to house OGM data. Raw .bnx files and analysis output files will be shared upon request.

## Supplementary Materials

The following supporting information can be downloaded at: https://www.mdpi.com/article/10.3390/cancers15194687/s1, Figure S1: SV/CNV plot representing structural variations (SVs) and copy number variations (CNVs) detected at least twice in samples from the cohort; Figure S2: Aneuploidy gains and losses of each chromosome with associated fCN values for each sample; Figure S3: Circos plots for all 10 cases with *IGH* rearrangements; Figure S4: Circos plots for all 10 cases characterized as HDM showing gains of odd-numbered chromosomes; Figure S5: de novo analysis (~250×) on samples 01, 33 and 44 with non-diploid genomes; Figure S6: CNV tracks for samples with a 1p12 deletion including the *NRAS* gene; Figure S7: OGM calls for gains of 1q including the *CKS1B* gene with associated fCN values; Figure S8: Genome view of the 8q24.21 chromosomal region for selected samples showing various abnormalities involving the *MYC* locus; Table S1: BED file of cancer genes used for OGM analysis; Table S2: Overall metrics for OGM samples; Table S3: Performance metric evaluation for optical genome mapping in myeloma.
