# Supplementary material for: Optical Genome Mapping Reveals the Complex Genetic Landscape of Myeloma
Source: Cancers (Basel). 2023 Sep 22;15(19):4687. doi: 10.3390/cancers15194687 (PMC10571866; doi:10.3390/cancers15194687)
Supplement: Supplementary file 1 [file cancers-15-04687-s001.zip › SupplementaryFile_Revision1_sept15-2023.pdf]

Supplementary Material

Table S1: BED file of cancer genes used for OGM analysis
See Excel File

Table S2: Overall metrics for OGM samples

Sample	DNA concentration (ng/µL)	N50 ≥150 kbp	Total DNA	Map rate	Effective coverage	NLV	PLV	Avg LD/100kbp
1	46.87	208.53	1518.47	87.6	414.41	7.7	6.95	16.5
2	29.4	219.75	1514.97	85.1	403.28	9.49	7.02	16.21
3	67.47	236.63	1509.75	81	382.42	12.43	6.1	15.6
5	44	240.38	1534.6	84.9	405.89	7.29	3.37	15.46
6	105	197.63	1241.32	73.5	282.8	16.38	2.08	16.38
7	66.96	213.38	1509.93	90.3	421.96	7.45	2.58	15.15
9	52	229.81	1538.69	80.1	384.87	8.79	5.01	15.97
12	121	244.88	1530.62	84.9	401.58	3.27	10.72	14.78
18	149.67	266.69	1541.63	81	392.52	8.55	4.99	15.96
24	29.53	189.75	1502.38	82.1	382.89	8.8	2.7	14.75
25	52.63	203.63	1513.02	81.6	384.52	6.53	2.65	15.42
30	78.07	242.95	1546.1	90.3	437.4	6.1	5.25	16.23
33	78.3	284.63	1528.61	92.8	445.86	6.4	6.33	16.41
37	49.1	293.63	1513.98	93.3	440.28	6.33	5.01	16.25
38	63.23	320.64	1561.51	93.8	456.89	6.34	5.89	16.05
41	74.33	342	1505.26	94.3	443.19	7.27	5.64	16.03
42	64.23	320.25	1519	93.1	443.13	6.36	8.24	16.69
43	65.8	242.25	1547.47	91.4	440.63	6.78	5.58	15.95
44	88.83	294.75	1506.44	93.7	442.11	7.99	5.91	16.1
45	32.23	313.5	1100.12	55.7	167.43	8.06	5.75	20.86

NLV= Negative Label Variance; PLV = Positive Label Variance; LD = Label Density
Ideal minimum quality values: N50 (≥150 kbp) ≥ 230 kbp; Total DNA = 1500; Map rate ≥ 70%; Effective coverage ≥ 300x; NLV < 15; PLV < 10; Avg LD = 14-17/100kbp
Values in red fall outside of the ideal minimum quality value

Table S3. Performance metrics evaluation for Optical Genome Mapping in myeloma.

Performance Criteria	Overall	*IGHr*	Deletions	*TP53* deletion	1p32 deletion	Gains	1q21 gain	1q21 amp
% Sensitivity (TP/(TP+FN))	100	100	100	100	100	85.7	100	66.7
% Specificity (TN/(TN+FP))	98.5	100	90.3	100	82.4	100.0	100	100
% PPV (TP/(TP+FP))	97.0	100	75	100	50	100	100	100
% NPV (TN/(TN+FN))	100	100	100	100	100	93.3	100	88.9
Accuracy (TP+TN/All Results)	99	100	92.5	100	85	95	100	90

TP = True Positive. TN = True Negative. FP = False Positive. FN = False Negative. PPV = Positive predictive value. NPV = Negative predictive value. Only data for which there was both a FISH and OGM result for all samples was included in this analysis.

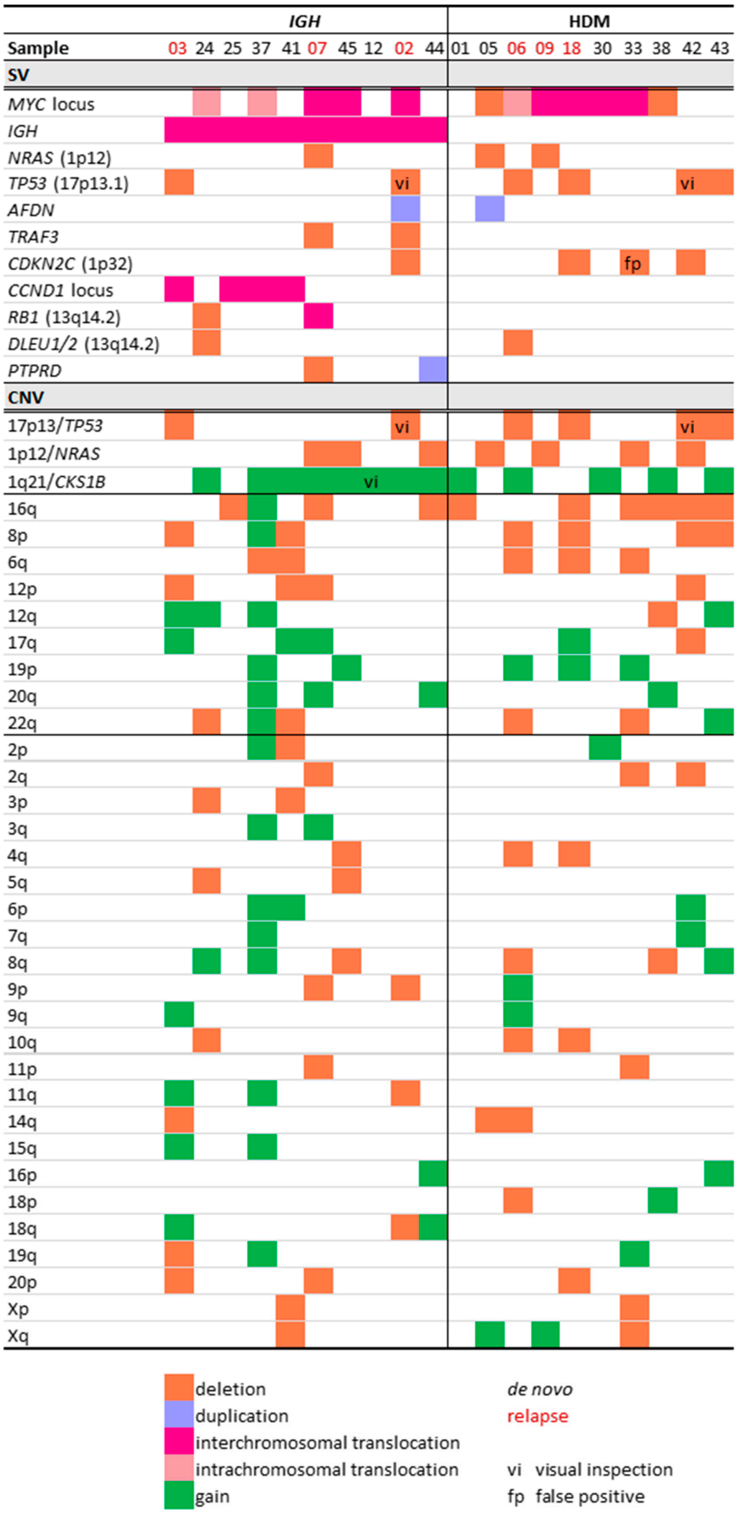

Figure S1: SV/CNV plot representing structural variations (SVs) and copy number variations (CNVs) detected at least twice in samples from the cohort. Each type of abnormality is represented by a different color. Samples are divided according to their primary genetic category (rearrangement of the *IGH* or hyperdiploidy). CNV were included in the figure for each chromosomal arm. regardless of the size of the CNV call. *De novo* myeloma samples are indicated in black and relapsed/refractory myeloma samples are indicated in red. vi: visual inspection; fp: false positive.

		Chromosome Gains																Chromosome Losses											
	**Sample**	**3**	**5**	**6**	**7**	**9**	**11**	**12**	**14**	**15**	**17**	**18**	**19**	**20**	**21**	**22**	**X**	**4**	**8**	**12**	**13**	**14**	**15**	**16**	**18**	**21**	**22**	**X**	**Y**
**HDM**	**01**		3.28	2.68	3.27	2.68			2.6	2.67	2.61		3.21						1.47		1.35				1.33	1.57	1.43	1.38	
	**05**	2.95	2.97			2.97	3.04			3.91			2.89						1.23										
	**06**	2.88			2.49		2.8			2.87																			
	**09**	2.93	2.94			2.99	3			3.93			2.85		3.77														
	**18**	2.97	2.91		2.97	3.02	3			2.96					2.96													1.03	
	**30**	3.37	3.37		3.39					4.48		3.39	4.28								1.19								
	**33**	2.63	2.56		2.65	2.63				2.69											1.35	1.35							
	**38**					3.02	4.08			3.02			2.88								0.99							0.99	
	**42**		3.4		2.86	3.39	3.4			3.39		3.37	3.36								1.88							1.26	
	**43**	3.02	2.55		2.54	2.87	2.54				3.38	2.94	2.96																
IGH	**02**																				1.03							0.99	
	**03**																				1.07			1.1			1.09		
	**07**																				1	1.02							
	**12**																				1.18	1.43					1.39		
	**24**																												
	**25**																											1.01	
	**37**																												
	**41**																				1.03								
	**44**										2.39							1.63		1.69	1.62								0.26
	**45**	2.57			2.54	2.47	2.58	2.56				2.52		2.38		2.38	1.27				1.56	1.45	1.41			1.40			0.13

Figure S2. Aneuploidy gains and losses of each chromosome with associated fCN values for each sample. Shades of green have been used to visualize fCN within the same range (<1. from 1 to 1.99; from 2 to 2.99; from 3 to 3.99 and above 4).

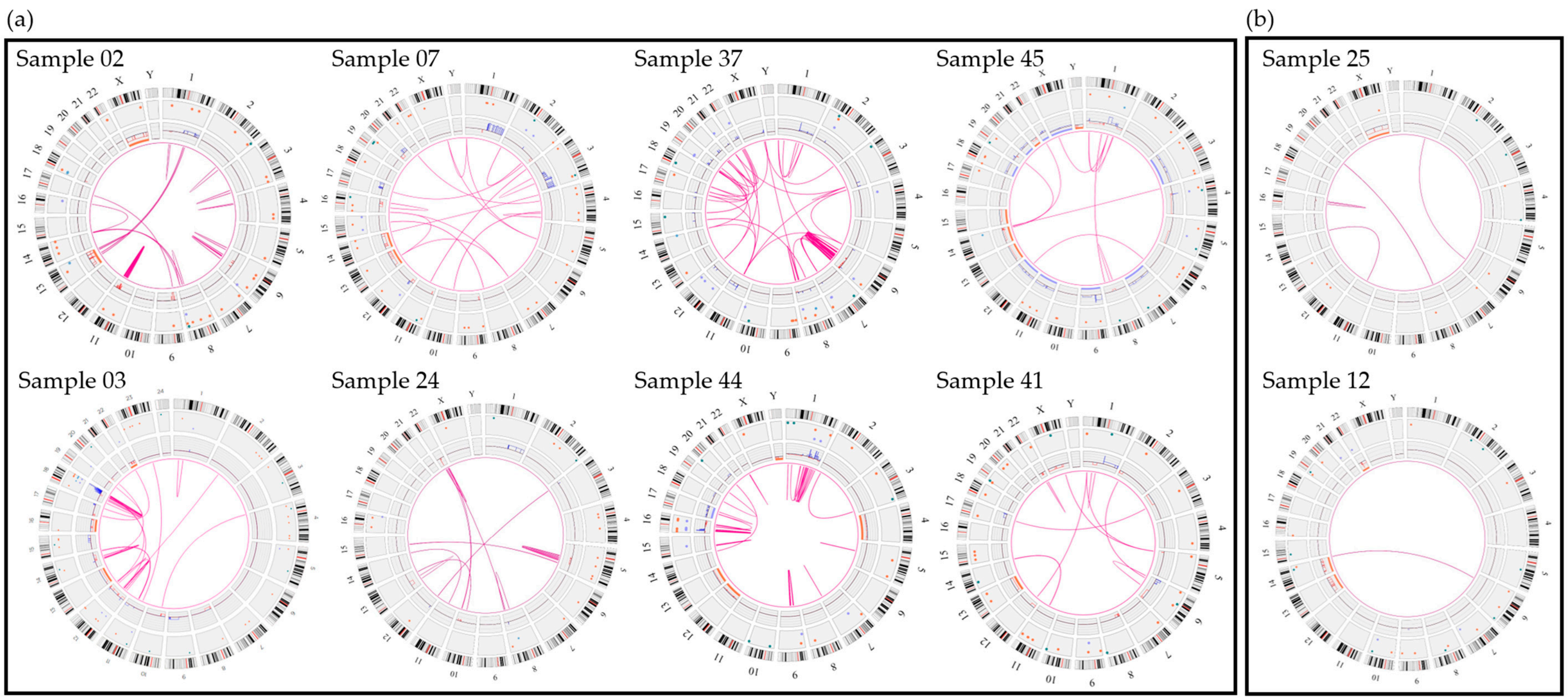

Figure S3. Circos plots for all 10 cases with *IGH* rearrangements. All cases presented with at least three abnormalities. thus meeting the definition of a complex genome according to criteria from the International System for human Cytogenomic Nomenclature (ISCN) 2020. A cut off value of more than 5 abnormalities was used to classify samples as simple or complex. (a) Complex genomes showing at least 5 chromosomal abnormalities. including gains and losses. (b) Simple genomes with 5 or less chromosomal abnormalities.

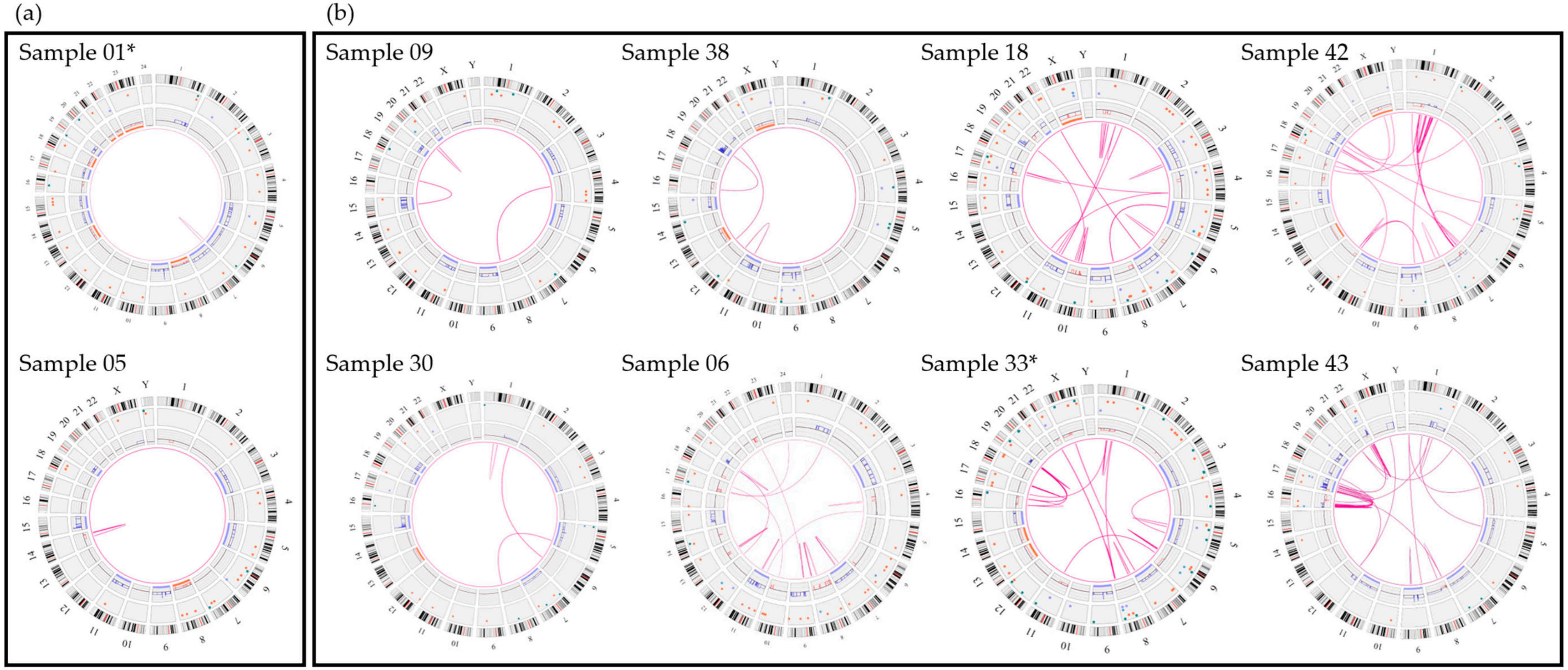

Figure S4. Circos plots for all 10 cases characterized as HDM showing gains of odd-numbered chromosomes. All samples showed complex genomes with at least three chromosomal abnormalities. as per the definition of ISCN 2020. (a) HDM samples with simpler genomes. characterized by less than three chromosomal rearrangements (excluding all aneuploidies). (b) HDM samples with more complex genomes. characterized by three or more chromosomal rearrangements (excluding aneuploidies). Asterisk denotes samples with non-diploid genomes.

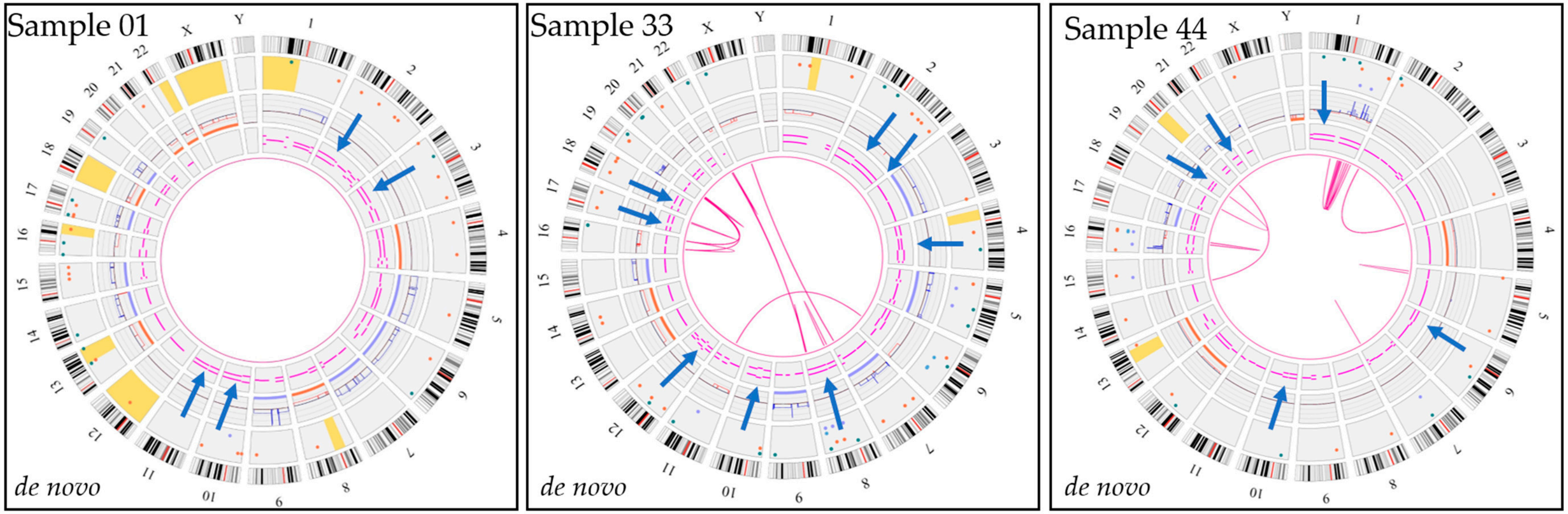

Figure S5. *de novo* analysis (~250X) on samples 01. 33 and 44 with non-diploid genomes showing consistent split in the VAF track (separated pink lines pointed by blue arrows) for most chromosomes without any associated CNV. SV or aneuploidy call (blue arrows). Chromosomal regions highlighted in yellow denote loss of heterozygosity (LOH).

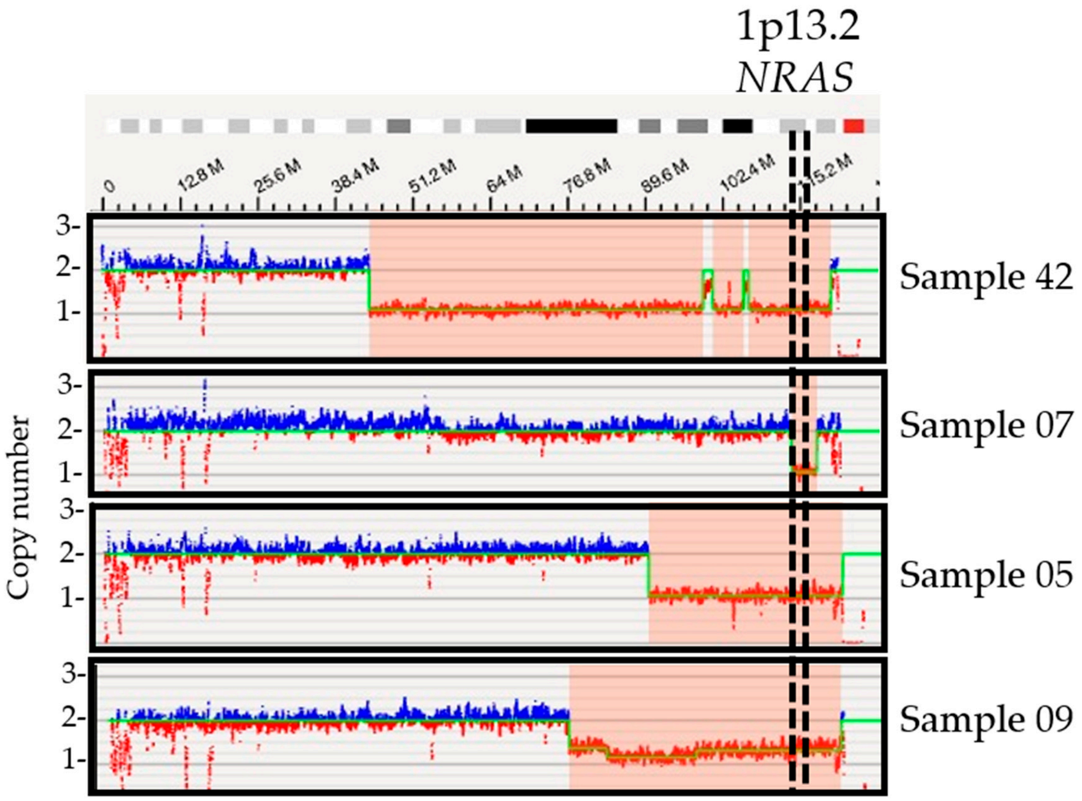

Figure S6. CNV tracks for samples with a 1p12 deletion including the *NRAS* gene.

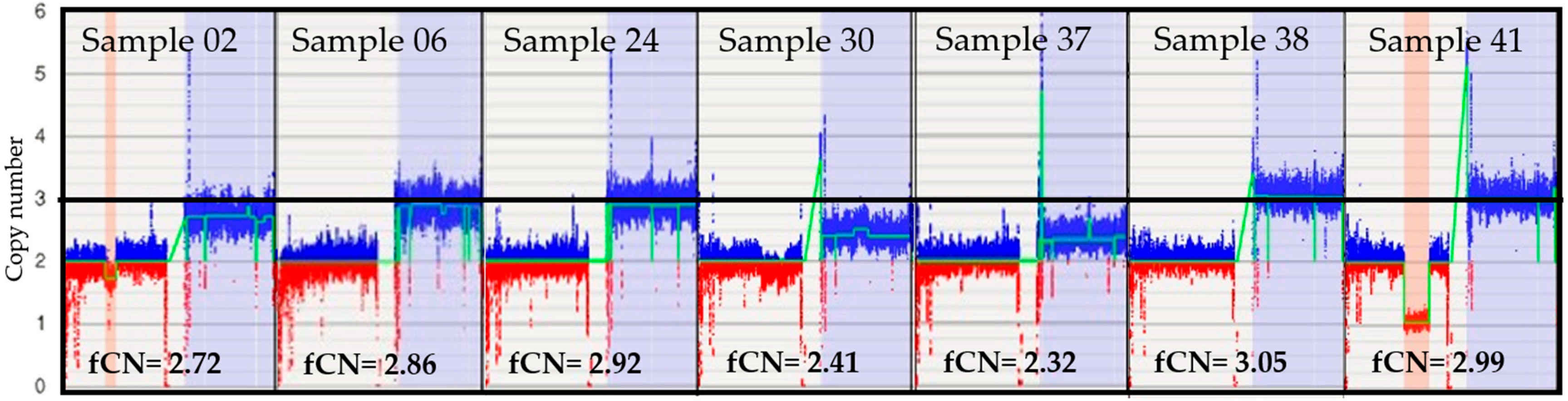

Figure S7. OGM calls for gains of 1q including the *CKS1B* gene with associated fCN values.

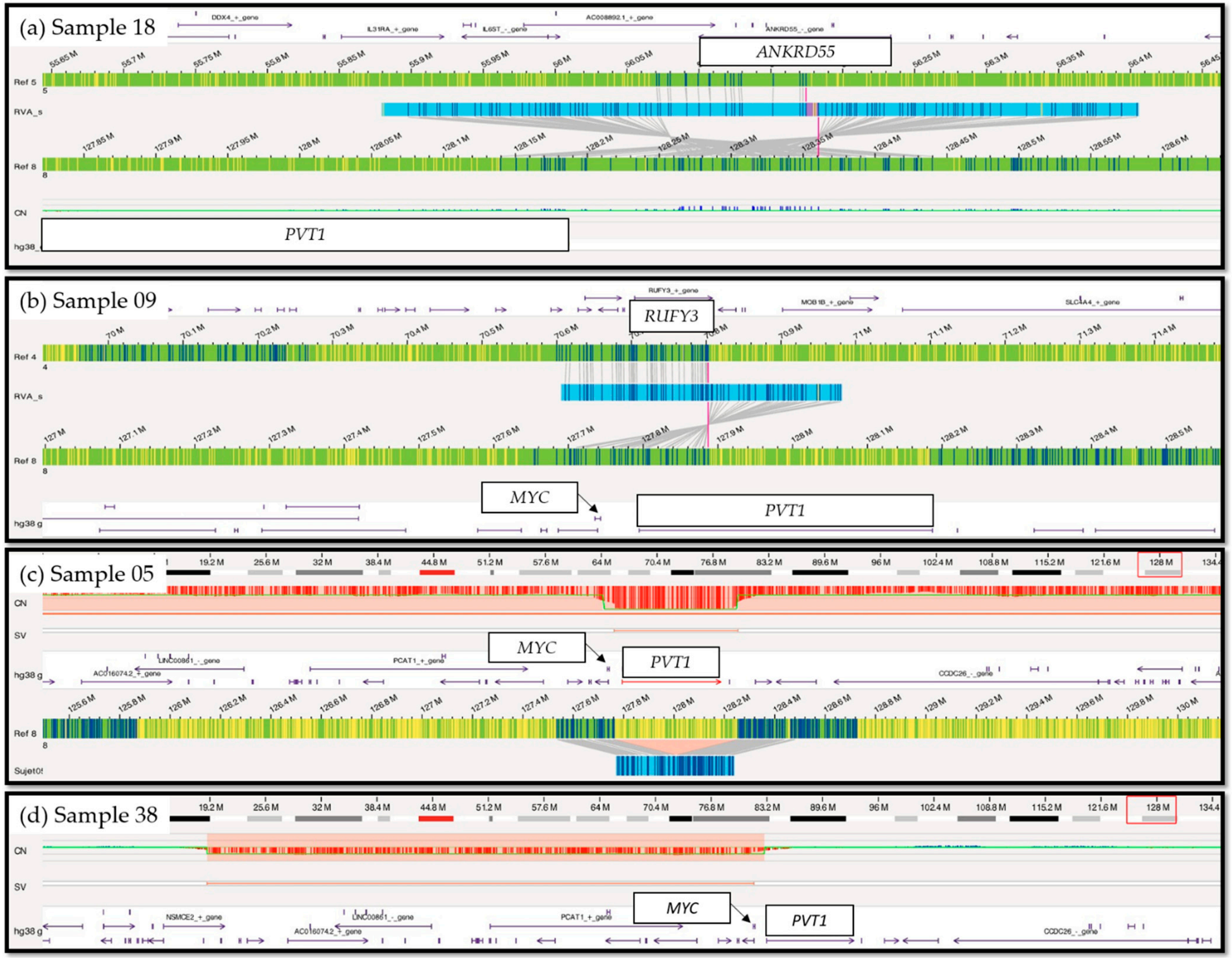

Figure S8. Genome view of the 8q24.21 chromosomal region for selected samples showing various abnormalities involving the *MYC* locus. (a) Sample 18 was identified by Access as an interchromosomal translocation involving chromosome 5. Consensus map shows an inversion and disruption of the 3′ region of the *PVT1* gene (8q24.21) and an insertion of sequences of the *ANKRD55* gene (5q11.2) within this inverted region. (b) Sample 09 was described as an interchromosomal translocation involving the chromosome 4. Consensus map shows an inversion of *PVT1* sequences fused to the *RUFY3* gene (4q13.3). (c) Genome view for sample 05 shows a complete monosomy of chromosome 8 as well as a targeted deletion encompassing the *PVT1* gene, thus leading to a biallelic deletion. (d) Genome view of the 8q24.21 for sample 38 showing a large-sized deletion encompassing the *MYC* gene and the 5′ region of *PVT1*.
